# Heterodimers of photoreceptor-specific nuclear receptor (PNR/NR2E3) and peroxisome proliferator-activated receptor-*γ* (PPAR*γ*) are disrupted by retinal disease-associated mutations

**DOI:** 10.1038/cddis.2017.98

**Published:** 2017-03-16

**Authors:** Joel Fulton, Bismoy Mazumder, Jonathan B Whitchurch, Cintia J Monteiro, Hilary M Collins, Chun M Chan, Maria P Clemente, Miguel Hernandez-Quiles, Elizabeth A Stewart, Winfried M Amoaku, Paula M Moran, Nigel P Mongan, Jenny L Persson, Simak Ali, David M Heery

**Affiliations:** 1 School of Pharmacy, University of Nottingham, Nottingham, UK; 2 Division of Clinical Neuroscience, School of Medicine, University of Nottingham, Nottingham, UK; 3 School of Psychology, University of Nottingham, Nottingham, UK; 4 School of Veterinary Medicine and Science, University of Nottingham, Nottingham, UK; 5 Department of Pharmacology, Weill Cornell Medicine, New York, NY, USA; 6 Division of Experimental Cancer Research, Department of Translational Medicine, Lund University, Clinical Research Centre, Malmö, Sweden; 7 Department of Molecular Biology, Umeå University, Umeå, Sweden; 8 Department of Surgery and Cancer, Imperial College London, London, UK

## Abstract

Photoreceptor-specific nuclear receptor (PNR/NR2E3) and Tailless homolog (TLX/NR2E1) are human orthologs of the NR2E group, a subgroup of phylogenetically related members of the nuclear receptor (NR) superfamily of transcription factors. We assessed the ability of these NRs to form heterodimers with other members of the human NRs representing all major subgroups. The TLX ligand-binding domain (LBD) did not appear to form homodimers or interact directly with any other NR tested. The PNR LBD was able to form homodimers, but also exhibited robust interactions with the LBDs of peroxisome proliferator-activated receptor-*γ* (PPAR*γ*)/NR1C3 and thyroid hormone receptor b (TRb) TR*β*/NR1A2. The binding of PNR to PPAR*γ* was specific for this paralog, as no interaction was observed with the LBDs of PPAR*α*/NR1C1 or PPAR*δ*/NR1C2. In support of these findings, PPAR*γ* and PNR were found to be co-expressed in human retinal tissue extracts and could be co-immunoprecipitated as a native complex. Selected sequence variants in the PNR LBD associated with human retinopathies, or a mutation in the dimerization region of PPAR*γ* LBD associated with familial partial lipodystrophy type 3, were found to disrupt PNR/PPAR*γ* complex formation. Wild-type PNR, but not a PNR309G mutant, was able to repress PPAR*γ*-mediated transcription in reporter assays. In summary, our results reveal novel heterodimer interactions in the NR superfamily, suggesting previously unknown functional interactions of PNR with PPAR*γ* and TR*β* that have potential importance in retinal development and disease.

Nuclear receptors (NRs) are a superfamily of gene regulators controlling a range of physiological processes and developmental pathways.^1^ Most NRs possess a zinc-finger DNA-binding domain (DBD) for target gene recognition and a ligand-binding domain (LBD) that facilitates homo- or heterodimeric interactions of NRs, agonist/antagonist responsiveness and transcriptional regulation via cofactor recruitment. Ligands control the activity of many NRs by inducing conformational changes in the LBD to favor coactivator or corepressor recruitment, although true orphan NRs appear to lack ligand regulation. NR cofactors contain amphipathic *α*-helices comprising signature LXXLL or LXXI/LXXXI/L motifs that dock with the LBD surface.^2, 3, 4, 5^ Although steroid receptors and some orphan NRs function principally as homodimers, many NRs including peroxisome proliferator-activated receptors (PPARs), RARs, VDR and TRs form heterodimers with RXRs. Heterodimer formation impacts on NR functions by altering their preference for specific DNA target sequences, as well as ligand response and interactions with cofactors.^6, 7, 8^ However, systematic studies of the dimerization properties of human NRs, in particular the orphan NRs, are incomplete.^9^

TLX and photoreceptor-specific NR (PNR) are members of the NR2E orphan receptor subgroup that are most closely related to the COUPTF (NR2F) and RXR (NR2B) subgroups.^10^ In mammals, TLX/NR2E1 is expressed in neurogenic regions of the brain and the retina, where it regulates neural stem cell maintenance and neocortex development. Deletion of the TLX gene in mice is associated with a pathological aggression (*fierce*) phenotype in addition to retinal dystrophy.^11, 12, 13, 14^ PNR/NR2E3 is required for photoreceptor development and function,^15, 16^ and its expression in adults is restricted to the outer nuclear layer of the neurosensory retina, where it functions to repress cone genes and commit photoreceptor precursors to rod cell fate.^17, 18^ A deletion within the NR2E3 gene in the rd7 mouse is associated with retinal degeneration,^19, 20^ whereas sequence variants in this gene in humans are associated with clinical phenotypes including enhanced S cone syndrome (ESCS), Goldmann–Favre syndrome, clumped pigmentary retinal degeneration and autosomal dominant or recessive retinitis pigmentosa.^21, 22^

PNR and TLX regulate gene expression through recruitment of corepressor complexes,^22, 23, 24^ and recently, we demonstrated that these NRs interact directly with BCL11A/B proteins,^25^ cofactors for the COUPTF/NR2F subfamily that function in globin gene switching and neurogenesis.^26, 27^ Indeed, we identified a novel motif (F/YSXLXXXL/Y) in BCL11A/B and NSD1 proteins that facilitates their direct and selective interactions with the NR2E/F subgroup.^25^ Although monomeric TLX can bind 5′-AAGTCA-3′ half-sites in the control regions of its target genes,^11, 28^ PNR bind direct repeats of 5′-ANGTCA-3′ sites separated by 1 bp (DR1) as homodimers.^15^ This suggests that PNR and TLX may have distinct dimerization capabilities, although this has not been investigated systematically. Here we describe a systematic analysis of PNR and TLX LBD interactions with other NRs and present evidence for novel heterodimeric interactions of PNR with PPAR*γ* and TR*β*.

## Results

### Distinct dimerization functions of the LBDs of human PNR and TLX

**Figure 1:**
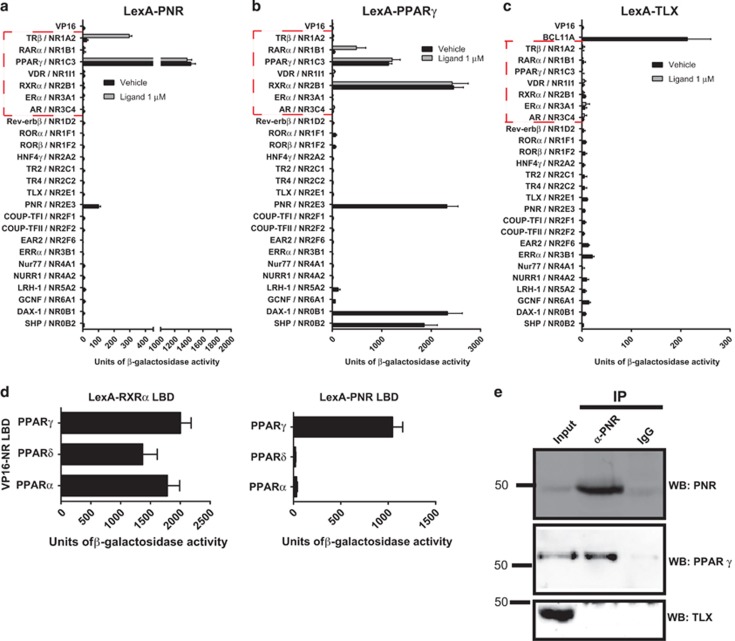
Novel heterodimerization interactions within the NR superfamily. (**a**–**c**) Yeast two-hybrid assays on *S. cerevisiae* L40 cell-free extracts co-expressing LexA–DBD fusion proteins containing the LBDs of PNR, PPAR*γ* or TLX, in combination with a panel of AAD-NR LBDs. A single representative experiment is shown, and the data represent the mean *β*-galactosidase activity of three independent clones, with error bars representing the S.D. For AAD-NR LBDs where a cognate ligand is known (i.e., those NRs within the boxed area), assays were performed on cells cultured in the presence and absence of cognate ligands, as described in Materials and Methods. Note that LexA-DBD-PPAR*γ* is activated by endogenous agonists in yeast. (**d**) Yeast two-hybrid assays showing the specific LBD interactions of PNR and PPAR*γ*, but not PPAR*α* or PPAR*δ*; the RXR*α* LBD is shown to interact with all PPARs. (**e**) Co-immunoprecipitation of endogenous PNR and PPAR*γ* proteins from retinal tissue lysate. Expression levels of PNR PPAR*γ* and TLX proteins in input samples are shown

We assessed the abilities of PNR and TLX LBDs to form homo- or heterodimers in yeast two-hybrid experiments, using a panel of human NR LBDs expressed as AAD fusion proteins.^25^ Reporter activities were established for transformed yeast clones cultured in the presence or absence of agonist ligands for the partner LBDs, if known. As shown in Figure 1a, the PNR hinge-LBD (131–410) construct was able to form homodimers in the Y2H assays, consistent with other studies using EMSA^16^ mammalian two hybrid^29^ or BRET in mammalian cells.^30^ Moreover, strong activation of the yeast reporter was observed when the LexA-PNR LBD was co-expressed with the VP16 AAD-fused LBD of PPAR*γ* (but not either alone), suggesting direct association of the LBDs of PNR and PPAR*γ*. Similar levels of reporter activity were observed in the presence or absence of the PPAR*γ* agonist rosiglitazone, indicating that in yeast cells, this interaction is not dependent on addition of exogenous agonist ligands. We also observed reporter activation (albeit weaker) indicating interaction between the LBDs of PNR and NR1A2/TR*β*, upon addition of cognate ligand (T3) (Figure 1a). This was confirmed using a near full-length VP16-TR*β*1 construct, which showed improved expression/stability in yeast. The reporter activity was induced approximately 20-fold in the presence of T3 (Supplementary Figure S1). Thus, PNR LBD can heterodimerize with the ligand-bound TR*β* LBD, another NR known to be involved in the development of photoreceptors in the retina.^31, 32^

We focused on characterization of the strongest interacting pair in the Y2H assays, that is, PNR and PPAR*γ*. To confirm this interaction, we performed two-hybrid assays in the reverse combination, that is, using LexA-PPAR*γ* LBD with the AAD-NR LBD panel. Our previous studies indicated that yeast contains endogenous agonists that stimulate strong interactions of PPAR LBDs with SRC1 and TRAP220 cofactors,^25, 33^ therefore it was only necessary to add exogenous ligands for the partner LBDs (indicated by the red boxes in Figures 1a and c). As shown in Figure 1b, the PPAR*γ* LBD displayed strong interactions with the LBDs of NR2B1/RXR*α*, NR0B1/DAX-1 and NR0B2/SHP consistent with other studies.^9, 34, 35, 36^ Homodimerization of the PPAR*γ* LBD was also detected, and similar levels of reporter activation were observed in the presence or absence of rosiglitazone, confirming activation of PPARs by endogenous ligands. Importantly, we also detected a strong interaction with PNR LBD, thus confirming dimerization of PPAR*γ* and PNR in Y2H assays. Interestingly, we noted a weak ligand-stimulated interaction of PPAR*γ* with the LBD of NR1B1/RAR*α*, which to our knowledge has not been reported previously, although it was not investigated further here.

We next tested the ability of the human TLX LBD to interact with the same set of NRs. As a control, TLX LBD displayed strong binding to the RID1 sequence of BCL11A comprising a FSXXLXXL motif as previously reported.^25^ However, we did not observe any evidence of interaction of the TLX LBD with itself or any other NR LBD tested in these assays (Figure 1c). This result supports previous studies suggesting that vertebrate TLX functions as a monomer.^11, 16, 37^

The ability of PNR to associate with PPAR*γ* LBD appeared to be specific for this paralog, as no interaction was observed with PPAR*α* or PPAR*δ* LBDs, whereas all three PPARs showed robust interaction with the LBD of RXR*α* in the absence of exogenous ligand (Figure 1d). We confirmed that the PNR hinge domain (or D region) was neither necessary nor sufficient for complex formation with PNR or PPAR*γ* LBDs (Supplementary Figure S2), supporting the hypothesis that the interactions involve LBD heterodimers. These results confirmed that PNR LBD dimerization was not dependent on the sequence corresponding to H1/H2 in other NR LBDs, consistent with the reported crystal structure of a truncated PNR LBD dimers lacking this sequence.^29^ GST pulldown assays detected interactions of full-length His-tagged PNR with the LBDs of PPAR*γ*, TR*β* and PNR, but not TLX (Supplementary Figure S3) albeit difficult to demonstrate ligand-stimulated effects in the *in vitro* assays, as reported in other studies.^38^ Taken together, our results suggest the existence of functional interactions of PNR with PPAR*γ* and TR*β*, as a consequence of LBD-mediated heterodimer formation. Interestingly, PPAR*γ*, TR*β*2 and PNR are each implicated in the development and function of mammalian retinal tissues^39, 40, 41^ although their direct interactions were not previously known.

To validate the existence of PNR/PPAR*γ* heterodimeric complexes, co-immunoprecipitation assays were performed on cell-free extracts of adult human retinal tissue. This tissue was found to express PNR, TLX and PPAR*γ* proteins as determined by western blotting (Figure 1e; inputs). PNR was readily immunoprecipitated from retinal extract, and western blots confirmed co-immuno-precipitation of PPAR*γ*, but not TLX (Figure 1e). This result indicates the existence of PNR/PPAR*γ* complexes in adult human retinal tissue. We also detected co-immunoprecipitation of endogenous PNR and PPAR*γ* proteins in the breast cancer cell line MDA-MB-468 (Supplementary Figure S4).

### Retinopathy-associated PNR mutations perturb interactions with PPAR*γ* and BCL11A

**Figure 2:**
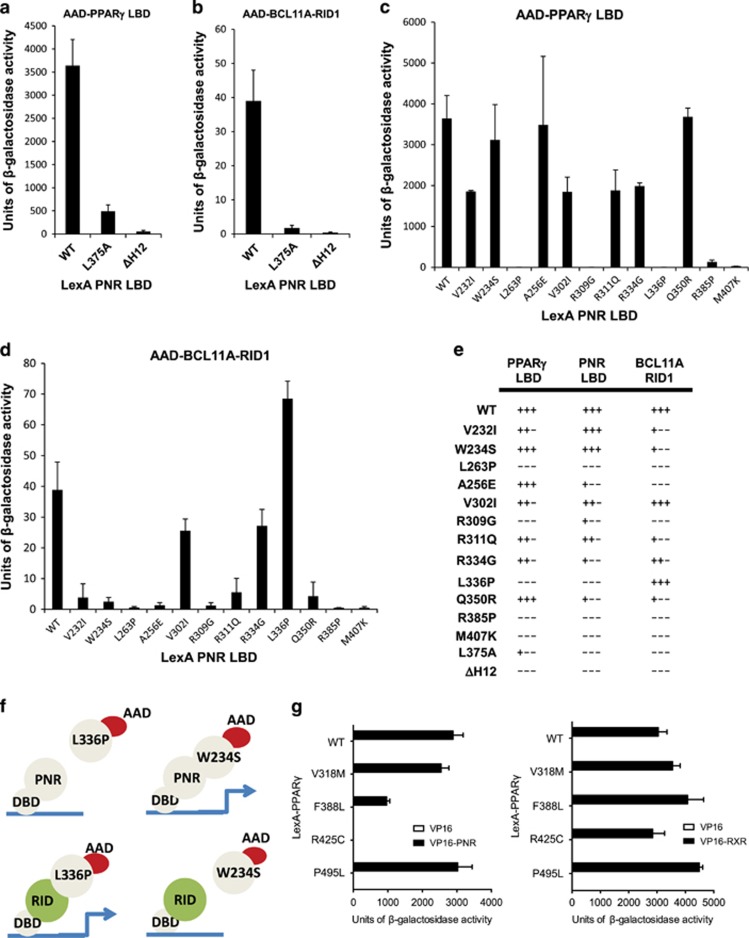
Disruption of PNR/PPARγ complexes by disease-associated mutations Yeast two-hybrid assays showing interactions of LexA-PNR LBD constructs as indicated in combination with (**a**) AAD -PPARγ LBD or (**b**) AAD-BCL11A 1-376 containing the RID motif. The L375A is designed to disrupt PNR dimerization via helix H10/11 and ΔH12 is a C-terminally truncated mutant lacking the AF2 helix. (**c**) Interaction of LexA-PNR LBD wild-type or ESCS-associated variants with AAD-PPARγ LBD or (**d**) AAD-BCL11A 1-376. (**e**) Qualitative summary of the effects of LBD variants on homodimer, heterodimer and corepressor interactions of PNR. (**f**) Schematic highlighting the differential effects of W234S and L336P on dimerization and cofactor binding by the PNR LBD in Y2H assays. (**g**) Two-hybrid interactions of LexA-PPARγ LBD wild-type or mutant constructs with VP16 AAD only (white bars) or AAD-PNR LBD or AAD-RXR*α* LBDs (black bars). Data are the average of triplicates and error bars represent S.D.

Crystal structures of the apo-LBDs of NR2E/F family members have been determined, including COUPTFII, TLX and PNR.^29, 42, 43^ Along with others, we have demonstrated that deletion of the AF2 helix perturbs the dimerization functions of the COUPTF-II LBD.^25, 43^ Therefore, to probe the molecular interactions between the PNR and PPAR*γ* LBDs in more detail, we generated a LexA-PNR LBD 192-399 mutant lacking the AF2 helix (ΔH12). In addition, we constructed LexA-PNR LBD containing the substitution mutation L375A. This residue lies within H10 and its substitution has been reported to impair homodimer formation of PNR *in vitro*.^29^ Western blots revealed similar expression levels of wild-type and PNR L375A construct in yeast (see Supplementary Figure S5). Deletion of the AF2 helix (ΔH12) disrupted the ability of the PNR LBD to form homodimers or heterodimers with the PPAR*γ* LBD (Figures 2a and e) as well as interactions with the RID domain of its cofactor BCL11A (Figures 2b and e). This is consistent with our previous observations for COUP-TFII LBD homodimers in similar assays.^25^ As in other NRs, the PNR AF2 sequence forms an amphipathic *α*-helix containing a LXXLL-like motif (MXXLLXXM), which may stabilize the LBD structure and cofactor binding via hydrophobic interactions. However, we did not detect any interaction of a H12 sequence itself with PPAR*γ* LBD (Supplementary Figure S2).

Replacement of L375 with alanine also strongly reduced the heterodimerization with PPAR*γ* LBD in two-hybrid assays (Figures 2a and e), consistent with the location of this leucine at the proposed interface of PNR LBD homodimers.^29^ The loss of PPAR*γ* LBD interactions suggests that similar molecular surfaces are involved in homodimeric and heterodimeric complexes of PNR, whereas the inability to bind the corepressor motif may be due to perturbation of the cofactor binding site, or failure of the mutant constructs to form LBD dimers in the assay.

Clinical studies have identified a range of variants in the NR2E3 gene coding sequence associated with human retinal disease. These substitution mutations can perturb the functionality of the encoded PNR protein in transcriptional activity, homodimer formation and binding of cofactors such as atrophin.^21, 44^ To assess how these sequence variants impact on the ability of PNR LBD to associate with PPAR*γ*, we generated LexA-PNR LBD constructs comprising the most commonly encountered ESCS-associated variants within this domain, namely V232I, W234S, A256E, L263P, V302I, R309G, R311Q, R334G, L336P, R385P and M407K. We also generated PNR Q350R, which is associated with Goldmann–Favre syndrome.^45^

Following co-transformation of L40 cells with AAD-PPAR*γ* LBD and either wild-type or mutant LexA-PNR LBD constructs, Y2H assays revealed that several of the ESCS-associated mutations strongly disrupted the interactions of PNR and PPARγ LBDs (Figure 2c). A qualitative summary is depicted in Figure 2e. Mutations that had a strongly detrimental effect on PNR/PPAR*γ* interactions were L263P, R309G, L336P, R385P and M407K. In contrast, W234S, A256E and Q350R generally displayed similar levels of reporter activity as the wild-type PNR LBD, suggesting that these substitutions do not substantially perturb the ability of PNR LBD to associate with PPAR*γ* in the assay. PNR LBDs containing ESCS-associated mutations V232I, V302I, R311Q and R334G retained PPAR*γ* binding in this assay, albeit at reduced levels as indicated by the reporter activities. Similar effects of these mutations were observed regarding formation of PNR LBD homodimer interactions, although A256E, R334G and Q350R were more detrimental to PNR homodimer formation (Figure 2e).

Several of the ESCS-associated mutations were also found to disrupt binding to the corepressor BCL11A RID1 sequence (Figure 2d), albeit with a distinct profile. In addition to L263P, R309G, R385P and M407K, the mutants W234S and Q350R showed strongly reduced interactions with the corepressor peptide, while maintaining strong dimerization capabilities. In contrast, L336P, which disrupted dimerization interactions, had no negative effect on the RID1 binding. This differential effect on the dimerization and cofactor binding functions of the PNR LBD is shown schematically in Figure 2f. In summary, our results indicate that ESCS-associated mutations in the NR2E3 gene may not only impair PNR homodimer formation and cofactor interactions, but also its ability to form heterodimers with PPAR*γ*.

Sequence alignments of the LBDs of the human NR2E/F family reveal that several of the residues linked to retinopathy variants are well conserved (Supplementary Figure S6). L263 (located in H5) is conserved amongst the NR2E/F family but is replaced by alanine in the closest homologous group, that is, the RXRs. R309 (H7) is also conserved, with lysine as the only variant observed within this group. L336 (located in the turn between H8 and H9) is replaced by phenylalanine in TLX, although extended sequence in this loop region may account for the inability of TLX LBD to dimerize. The H10 residue R385 is not conserved as it is replaced with T,V, L or C residues in the other members of the group, whereas M407 in the AF2 helix is conserved among this subgroup of NRs.

We mapped the positions of variant residues using the crystal structure of the PNR LBD (a.a. 217–410) homodimer (PDB: 4LOG).^29^ The structure indicates that L263 is located in helix H5 at the core of the LBD and is likely to stabilize the LBD through multiple hydrophobic contacts (Supplementary Figure S7A). Thus, insertion of proline at this position may disrupt the LBD architecture and have catastrophic effects on LBD functions. Another proline substitution involves residue R385 located within H10/11, which forms the major dimer interface between LBDs. R385 is located within the more C-terminal portion of H10/11 (ref. 29) (Supplementary Figure S7B). In one of the PNR LBD monomers, R385 makes polar contacts with E299 (Supplementary Figure S7C), but shows an alternative conformation in the partner LBD. Although this residue is not well conserved in the NR2E/F subfamily, proline substitution is likely to perturb the structure because of steric clashes.

Interestingly, the PNR LBD L336P mutant maintained strong binding to BCL11A peptide in our assays, indicating at least partial function. This residue lies in the loop between H8 and H9 (Supplementary Figures S6 and S7A) and despite predicted steric clashes, the homology model suggests proline may be accommodated without perturbing the overall LBD structure (Supplementary Figure S7E). We superimposed the structures of a single LBD in the PNR dimer (4LOG) with the monomeric LBD of TLX/NR2E1 in complex with atrophin cofactor peptide (PDB: 4XAJ)^42^ (Supplementary Figure S9). Substantial overlap between the two structures was observed and the alignment revealed that the LXXLXXY cofactor peptide binding site is spatially distant from the L336P. Assuming that cofactor peptides can associate with monomeric LBDs in the Y2H assays, this provides a plausible explanation for the differential effect of L336P on cofactor binding and dimerization.

Residue R309 is located within H7 and potentially makes intramolecular polar contacts with L373, and intermolecular polar contacts with E332 in the partner LBD (Supplementary Figure S7D). The M407K mutation, which lies within the AF2 helix (H12), disrupted formation of PNR/PNR and PNR/PPAR*γ* LBD complexes in Y2H experiments (Figures 2c and e). Consistent with this, deletion of the AF2 helix had a similar effect on the formation of these complexes (Figures 2a and e). However, it is unclear from the currently available ‘autorepressed' structure how M407K impacts on LBD dimerization.

### The PPAR*γ* R425C mutation disrupts interaction with PNR

Rare sequence variants in the PPAR*γ* LBD, such as V318M, F388L, R425C and P495L, are associated with familial partial lipodystrophy type 3. These mutations have been reported to have differential effects on PPAR*γ* such as impairment of transcriptional activity, ligand binding, dimerization or corepressor dissociation,^46, 47^ and thus are generally associated with loss of function. We generated AAD-PPAR*γ* LBD mutants to assess the effects on association with wild-type PNR LBD (Supplementary Figure S5B). Interestingly, V318M, F388L and P495L mutants were able to induce reporter activation to a level comparable to the wild-type PPAR*γ* LBD, indicating robust interaction with PNR LBD, although in some replicate assays V318M showed a reduced interaction (data not shown). In contrast, the R425C mutant was significantly impaired in its ability to bind PNR LBD (Figure 2g), whereas all the PPAR*γ* constructs retained the ability to interact with RXR*α* LBD (Figure 2g). Examination of the crystal structure of the full-length PPAR*γ*/RXR*α* complex in association with DNA^48^ reveals that the R425 residue (which lies within a loop between helices H8 and H9) is positioned close to the interface between PPAR*γ* and RXR*α* LBDs (see Supplementary Figure S8). The sidechain of R425 makes polar contacts with E352 in H4/5 of the PPAR*γ* LBD, but does not appear to contact RXR*α* LBD directly.^38^ Thus, a pathogenic variant in the PPAR*γ* LBD associated with partial lipodystrophy can potentially impact on the formation of PPAR*γ*/PNR heterodimers.

### NR2E3/PNR suppresses PPAR*γ*-mediated reporter activation

**Figure 3:**
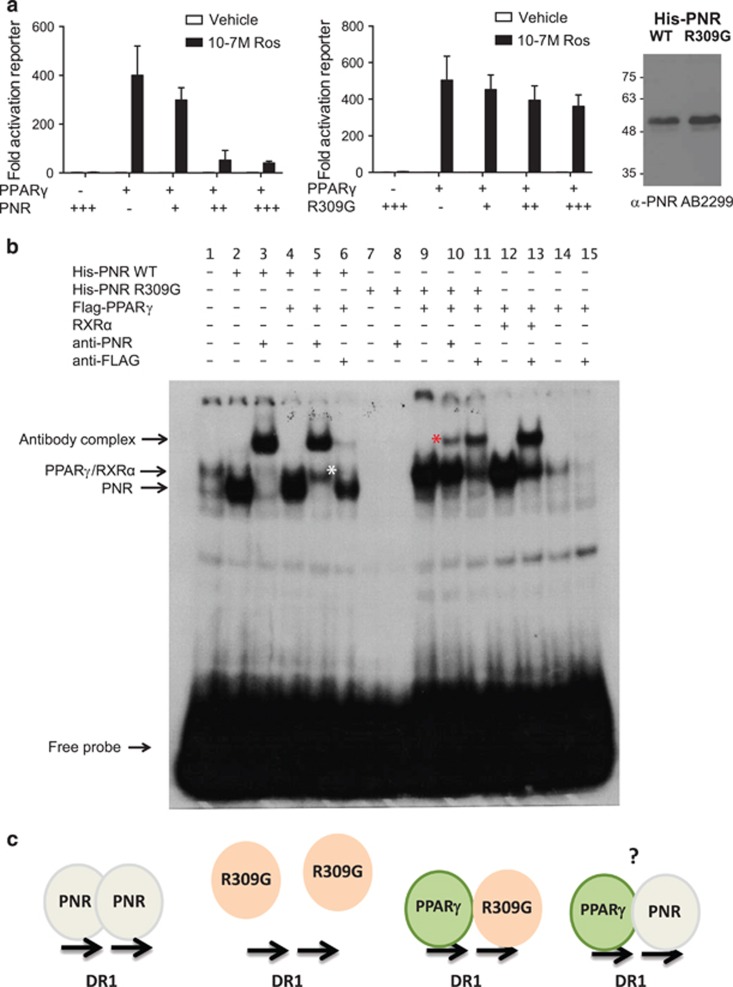
Repression of PPAR*γ*-mediated transcription by PNR. (**a**) Reporter assays showing ligand-dependent activation of a 3xPPRE-Luciferase by PPAR*γ* in transiently transfected U2OS cells and the effect of co-transfection of PNR wild-type and R309G expression vectors. Luciferase activity was normalized to the co-transfected *β*-galactosidase activity. Reporter activity in transfected cells was determined following 24-h exposure to 10^−7^M rosiglitazone or vehicle as indicated. Data show mean luciferase values from triplicates assays, and error bars indicate the S.E.M. The right panel is a western blot showing detection of wild-type and R309G His-PNR proteins expressed in transfected cells, detected with anti-PNR antibody. (**b**) EMSA assays and antibody supershifts showing binding of PNR wild-type or PNR (R309G), FLAG- PPAR*γ* or RXR*α* to a DR1 element in the combinations as indicated. Double-stranded DNA probes (end labeled with *γ*32P) were incubated with cell-free extracts of HEK293 cells expressing recombinant NR proteins, or mock transfected as indicated (as described in Supplementary Materials and Methods). Free probes and specific NR/DNA and antibody complexes are indicated on the image. The white asterix indicates a complex containing PPAR*γ* that is shifted by the anti-FLAG antibody, the red asterix indicates complexes containing both PPAR*γ* and PNR(R309G). (**c**) Schematic representation depicting the proposed interactions of PNR and PNR(R309G) complexes with the DR1 probe

To examine the functional implications of PNR interaction with PPAR*γ*, we carried out mammalian cell reporter assays. As in other studies,^38^ a reporter gene controlled by a basal promoter with three copies of a DR1 element 5′-AGGTCAnAGGTCA-3′ (3xPPRE-tk-Luc) was strongly activated by co-transfected FLAG-PPARγ in the presence of rosiglitazone (Figure 3a). Co-transfection with increasing amounts of PNR expression plasmid resulted in a dose-dependent repression of PPAR*γ*-activated reporter activity. However, this repression was not observed when the ESCS variant PNR R309G was co-expressed with PPAR*γ* under the same conditions. Western blots confirmed equal expression of the WT and R309G proteins (Figure 3b). These findings are consistent with reports that PNR can repress some of its gene targets,^15, 17^ potentially including PPAR*γ*-regulated genes as both NRs can bind DR1 elements. The results also show that the PNR R309G variant negatively impacts on the ability of PNR to repress PPAR*γ*-mediated transcription.

To explore possible mechanisms underlying the repression of PPAR*γ* reporter by PNR, we performed EMSA assays. HEK293 cells were transfected with expression vectors for PNR, FLAG-PPAR*γ* or RXR*α* and nuclear extracts prepared from the transfected cultures were admixed with a radiolabeled double-stranded DR1 probe containing a consensus PPAR response element (PPRE) (5′-nnnnAGGTCAaAGGTCAnn-3′). As shown in Figure 3b, in mock-transfected extracts, several bands were observed because of association of endogenous proteins with the DR1 probe (lane 1), which may be endogenous NRs. However, western blots did not detect any endogenous PNR proteins in HEK293 cells (data not shown). Exogenously expressed wild-type PNR was found to associate strongly with the DR1 probe, and could be supershifted by addition of PNR-specific antibody (compare lanes 2 and 3). In contrast, PNR309G was unable to associate with the DR1 probe, as no shifts were observed in the presence or absence of antibody (lanes 7 and 8) consistent with a previous study.^21^ Co-expression of FLAG-PPAR*γ* with PNR resulted in the formation of an additional second complex of higher molecular weight, albeit of lower intensity and partially masked by the PNR complex (lane 4). Addition of anti-PNR antibody caused a supershift of the PNR complex (lane 5), whereas FLAG antibody supershifted the remaining higher molecular weight band (lane 6), indicating this higher molecular weight complex contains FLAG-PPAR*γ*. We suggest that the FLAG-PPAR*γ* complex (indicated by a white asterix in lane 5) is probably formed by dimerization with endogenous RXRs, which are required for PPARs to bind DNA efficiently. Consistent with the hypothesis that formation of PPAR*γ*/DR1 complexes is limited by the availability of endogenous RXRs in the extracts, co-transfection of a RXR*α* and FLAG-PPAR*γ* expression vectors resulted in a much stronger shift of the probe because of binding of PPAR*γ*/RXR*α* heterodimers (compare lane 12 with 14 and 5), as confirmed by supershift with the FLAG antibody (lanes 13 and 15).

Interestingly, although PNR309G alone failed to give a detectable shift of the PPRE DR1 probe (lanes 7 and 8), co-expression with FLAG-PPAR*γ* yielded a strong complex of similar molecular weight to transfected PPAR*γ*/RXR*α* complexes (lane 9). This complex (indicated by a red asterix; Figure 3b) could be supershifted by either anti-PNR (lane 10) or anti PPAR*γ* antibodies (lane 11). This result suggests that while defective homodimerization of the PNR (R309G) variant compromises its ability to associate with a DR1 response element *in vitro*, (note: there is no endogenous wild-type PNR) it may be capable of forming heterodimeric associations with PPAR*γ*, at least *in vitro*. How this is achieved, and whether it requires DNA-binding activity of the PNR variant remains to be established.

**Figure 4:**
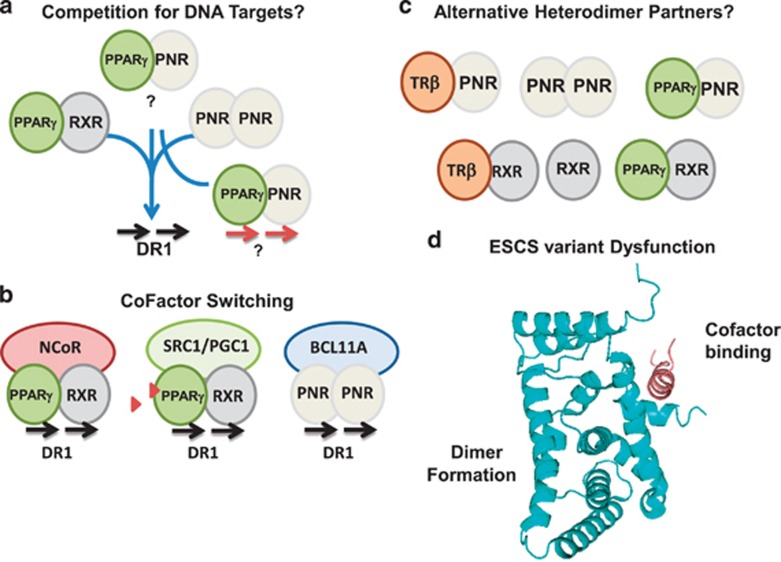
Potential functional interactions of PNR complexes Schematic model showing (**a**) proposed competition for DR1 type DNA targets; (**b**) possible role of PNR and PPARγ complexes in cofactor switching. Red triangles denote agonist ligands. (**c**) Novel heterodimeric complexes involving PNR and possible competition with TR*β*/RXR and PPAR*γ*/RXR heterodimers for dimeric partners. (**d**) Homology model of the PNR LBD (4LOG) indicating the distinct surfaces involved in dimerization and cofactor binding. The cofactor peptide is from a superimposed alignment of the TLX LBD in complex with atrophin corepressor peptide (4XAJ)^42^

In summary, as depicted schematically in Figure 4, we conclude that PNR homodimers and PPAR*γ*/RXR*α* heterodimer complexes can assemble independently on DR1 sequences, and therefore may compete for binding to such sites *in vivo* (Figure 4a). Both dimers bound strongly to canonical PPRE (5′-AGGTCAaAGGTCA-3′) (Figure 3b) or to a PNRE (5′-AAGTCAaAAGTCA-3′) (data not shown) probes in the EMSA assays. This may facilitate the assembly of different NR/ cofactor complexes on target genes (Figure 4b) in a tissue-specific or temporally regulated fashion. Our study provides evidence for novel heterodimeric interactions of PNR, PPAR*γ* and TR*β*, which may have important implications for understanding their roles in disease.

## Discussion

Although a subset of ligand-binding NRs have been well characterized, the dimerization capabilities of most orphan NRs have not been systematically studied. Here we used Y2H and other assays to assess the LBD dimerization capabilities of orphans PNR and TLX, which are predominantly expressed in retinal and CNS tissues, respectively. TLX LBD displayed no ability to form dimers with itself or other NRs, consistent with suggestions that TLX functions as a monomer.^11, 28^ Although a recent crystal structure of a modified TLX LBD bound to a corepressor peptide revealed a homodimeric LBD complex, we note that it was necessary to modify nine residues within the TLX LBD to resemble PNR surface residues, in addition to removing the H1 and H2 helices to obtain crystals.^42^ Our experiments failed to detect any evidence of dimer formation by TLX LBD, although it readily bound to a corepressor motif, in agreement with studies suggesting that vertebrate TLX functions primarily as a monomer.^37, 49^

### Novel PNR heterodimer complexes

In addition to confirming PNR LBD homodimerization in our Y2H assays, we observed novel heterodimeric interactions of PNR with PPAR*γ* and TR*β*. Although PNR/TRβ association was ligand dependent in yeast, PPAR*γ*/PNR dimers formed independently of exogenous ligand, likely due to the presence of endogenous PPAR agonists in yeast.^2, 25, 50^ These heterodimer interactions required the integrity of the AF2 helix, and were disrupted by mutations associated with human disease, such as ESCS (PNR) or lipodystrophy (PPAR*γ*) variants.

Like PNR, TR*β*2 functions in photoreceptor development,^41^ and its disruption in mouse results in failure to express M opsins, leading to a lack of M cones and an excess of S cone photoreceptors.^32^ In addition to forming dimers with RXRs and itself, TR*β* LBD has been reported to dimerize with Ear2/NR2F6^51^ and estrogen receptor ER*α*/NR3A1^52^ although interaction with NR2E3 has not been reported previously. However, the paralog TR*α* has been reported to heterodimerize with COUPTFs^53^ showing a precedence for TR interactions with the NR2E/F subfamily. Thus, direct interactions of PNR and TR*β*2 may contribute to the regulation of genes that determine photoreceptor fate.

Consistent with our findings, we note that PPAR*γ* is reported as a PNR-interacting protein in the BioGrid database, based on global mammalian two-hybrid screen data deposited by the FANTOM /RIKEN consortium.^36^ Although PNR and Reverb*α*/NR1D1 were reported to co-regulate retinal genes,^44, 54^ this involved interactions via DBD and hinge regions of both NRs, rather than the LBDs.^55^ Although we were unable to express a stable NR1D1 LBD in our assays, we did not detect an interaction of PNR with the paralog Rev-erb*β*/NR1D2 (Figure 1a), even in the presence of exogenous heme (data not shown), a ligand for Reverbs.^56, 57^

### PNR interaction with PPAR*γ*: a potential role in ocular disease?

Several NRs including PNR, TR*β*2, RORs, TLX, RXRs, RARs, ERRs, COUPTFs and Reverbs are implicated in retinal development and function.^41^ Interestingly, PPARs are also implicated in ocular diseases, including retinopathies, diabetic retinopathy and age-related macular degeneration.^58^ Therefore, our evidence for novel heterodimeric interactions of PNR and PPAR*γ* indicates a potentially important intersection of these regulators in retina or other tissues. PPAR*γ* is expressed in rodent neuroretina and retinal pigment epithelium, and several studies have indicated that PPAR*γ* agonists have protective effects for neuroretinal damage in rodent models.^40, 59, 60^ PPARγ has been proposed as a therapeutic target in ocular disease, because of its roles in the regulation of oxidative stress and suppression of VEGF-mediated neovascularization.^39, 40, 61, 62, 63^ In proliferative diabetic retinopathy, thiazolidinediones can ameliorate neovascularization by suppressing the expression of VEGF and FLT1.^60, 64^ Interestingly, FLT1 is also a target for regulation by PNR.^65^

In addition to their direct physical interaction, PNR homodimers and PPAR*γ*/RXR*α* heterodimers associate with DR1 elements *in vitro*,^15^ and we have demonstrated that both can bind canonical PPRE (Figure 3b) or PNRE elements (data not shown) with similar efficacy. Indeed, PNR was able to repress PPAR*γ*-mediated activation of a PPRE reporter (Figure 3a). However, the DNA-binding preferences and potential gene targets of PNR/PPAR*γ* heterodimers remains to be determined and warrants further investigation.

### Effects of ESCS-associated mutations on PNR function

Our results indicate that disease-associated LBD mutations L263P, R309G, L336P, R385P and M407K have detrimental effects on PNR function, in good agreement with previous studies that have assessed PNR functionality with regard to homodimer formation or binding of PNR to CRX, Rev-erbs, atrophin, DNA or reporter gene repression assays.^21, 30, 44, 66^ Fradot *et al.*^66^ showed that PNR carrying R385P or M407K mutations lost the ability to repress a reporter gene, whereas W234S and R311Q retained DNA-binding, homodimerization and reporter gene repression functions. Although a GAL4-PNR LBD fusion carrying R309G exhibited reduced repressor activity compared with wild-type, this mutant was not assessed in terms of DNA binding or in the context of full-length PNR. Our data show that the R309G mutation perturbs both homo- and heterodimerization of the PNR LBD in Y2H assays (Figures 2c and e) and impairs the ability of full-length PNR to bind DR1 and repress PPAR*γ*-mediated reporter activation (Figures 3a and b). Intriguingly, overexpression of PPAR*γ* rescued the ability of PNR (R309G) to associate with DR1 complexes in EMSA experiments (Figure 3b). In contrast to the Y2H assays, full-length PNR proteins in the EMSA assays may help stabilize heterodimer interactions in the EMSA assays.

Kanda and Swaroop^21^ reported that many NR2E3 clinical variants impact on the subcellular localization of PNR, resulting in increased localization to the cytoplasm to variable extents, including W234S, A256E, L263P, R309G, R311Q, R334G, L336P, R385P and M407K. This showed at least partial correlation with loss of DNA-binding ability in EMSA assays, with only V302I, R311Q, R334G and M407K retaining DNA-binding activity but displaying reduced interaction with CRX/NRL. PNR activation of a reporter gene was also reduced by approximately twofold for most mutations with the exceptions of V232I, W234S, R311Q and R334G.

Our Y2H data showed that reporter activation because of PNR LBD homodimerization was an order of magnitude lower than with PPAR*γ* (Figures 1, 2 and data not shown). Although V232I and W234S did not show any decreased interaction compared with wild-type, qualitatively, most mutations perturbed PNR LBD homodimer formation in yeast, although V302I and R311Q had only moderate impact. Moreover, our data revealed differential effects on binding of the RID motif of BCL11A (Figure 2d), a potent corepressor for the NR2E/F family.^25^ Although L263P, R309G, R385P and M407K disrupted BCL11A binding as may be expected, the mutants W234S and Q350R also showed strongly reduced interaction with the corepressor peptide. The Q350R mutation did not hamper binding to PPAR*γ*, consistent with its location away from the dimerization interface. It is therefore possible that this mutation impacts on cofactor recruitment. In contrast, L336P, which disrupted PNR LBD dimerization interactions, had no negative effect on the RID1 binding, highlighting that different variants may invoke distinct functional impairments. Taken together, these findings (summarized in Figure 4) suggest that disease-associated variants in the NR2E3 gene not only impair LBD homodimer and cofactor interactions, but also impair heterodimer interactions with PPARγ or other NRs.

Although there was generally good agreement between our studies and previous reports, different experimental approaches or poor stability of mutant proteins may produce discrepancies regarding the impact of different mutations. Several groups^22, 29^ have reported instability of PNR mutants, and we also observed some evidence of this in some yeast clones expressing R309G, R311Q and R334G constructs. Therefore, we were careful to confirm equivalent expression by western blots for any transformants included in reporter assays (Supplementary Figure S5).

### Mutations in PPARγ and lipodystrophy

PPAR*γ* has central roles in metabolic homeostasis through the regulation of lipid and glucose metabolism. With the exception of the P12A polymorphism, genetic variants in the PPARγ gene are rare and LBD variants have been associated with familial partial lipodystrophy type 3, leading to loss of fat tissues at the extremities combined with insulin resistance and type 2 diabetes.^46^ These mutations generally result in loss of function and reduced transcriptional activity of PPAR*γ*. The variants V318M, F388L and P495L reside in helices H3, H7 and H12, respectively, and have been reported to impact on LBD functions other than dimerization.^46^ Our data reveal that the R425C variant also has a detrimental effect on PPAR*γ* heterodimer interactions with PNR, whereas RXR*α* binding was not significantly affected. Interestingly, retinal changes are sometimes observed in patients with acquired partial lipodystrophy.^67, 68^ Crystal structures suggest that R425C does not participate in direct contacts with the RXR*α* LBD,^48^ although it is located close to the heterodimer interface, and therefore may have a role in contacting the PNR LBD. Therefore, it is interesting to speculate on whether perturbation of PNR/PPAR*γ* heterodimers by genetic variants may have a role in retinal changes in partial lipodystrophy disorders.

In summary, although the major known function of PNR in mammals is to orchestrate the photoreceptor differentiation, PNR orthologs in frogs and fruitflies also function in the pineal gland development, reproduction and circadian clock function.^69, 70^ Anatomical profiling has detected low level NR2E3 expression in the enteric tract and in seminal vesicles^71^ suggesting possible PNR functions in other tissues. Moreover, cell line expression studies indicate co-expression of PNR and PPARγ in cells of tumor origin. Thus, PNR and PPAR*γ* may converge to regulate genes in response to developmental or pathogenic cues.

## Materials and methods

Detailed Materials and Methods for this study are available in the online Supplementary Material.

## Supplementary Material

Supplementary Figure Legends

Supplementary Material and Methds
