# Supplementary material for: Heterodimers of photoreceptor-specific nuclear receptor (PNR/NR2E3) and peroxisome proliferator-activated receptor-γ (PPARγ) are disrupted by retinal disease-associated mutations
Source: Cell Death Dis. 2017 Mar 16;8(3):e2677–. doi: 10.1038/cddis.2017.98 (PMC5386588; doi:10.1038/cddis.2017.98)
Supplement: Supplementary Figures [file cddis201798x1.pdf]

Figure S1

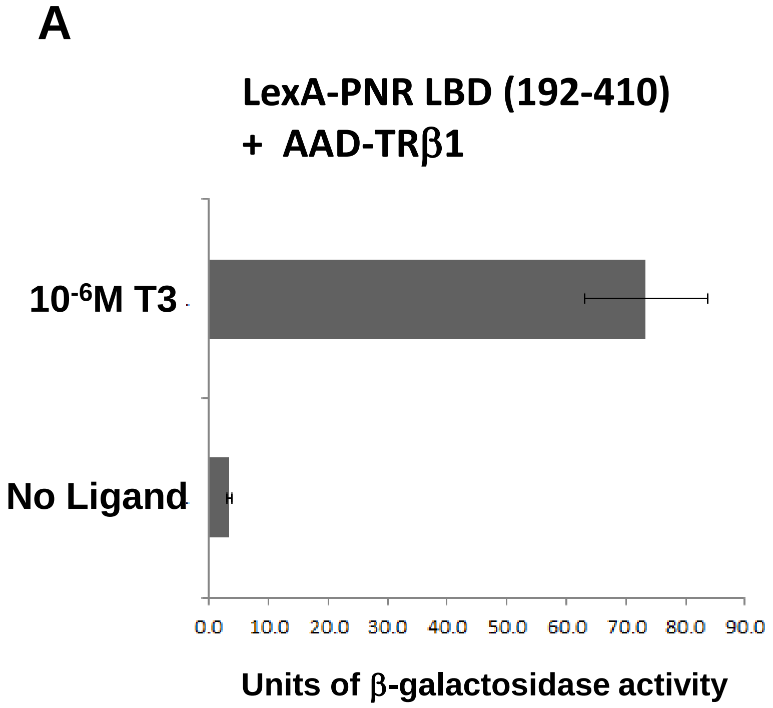
A
LexA-PNR LBD (192-410)
+ AAD-TRβ1
10-6M T3
No Ligand
0.0
10.0
20.0
30.0
40.0
50.0
60.0
70.0
80.0
90.0
Units of β-galactosidase activity

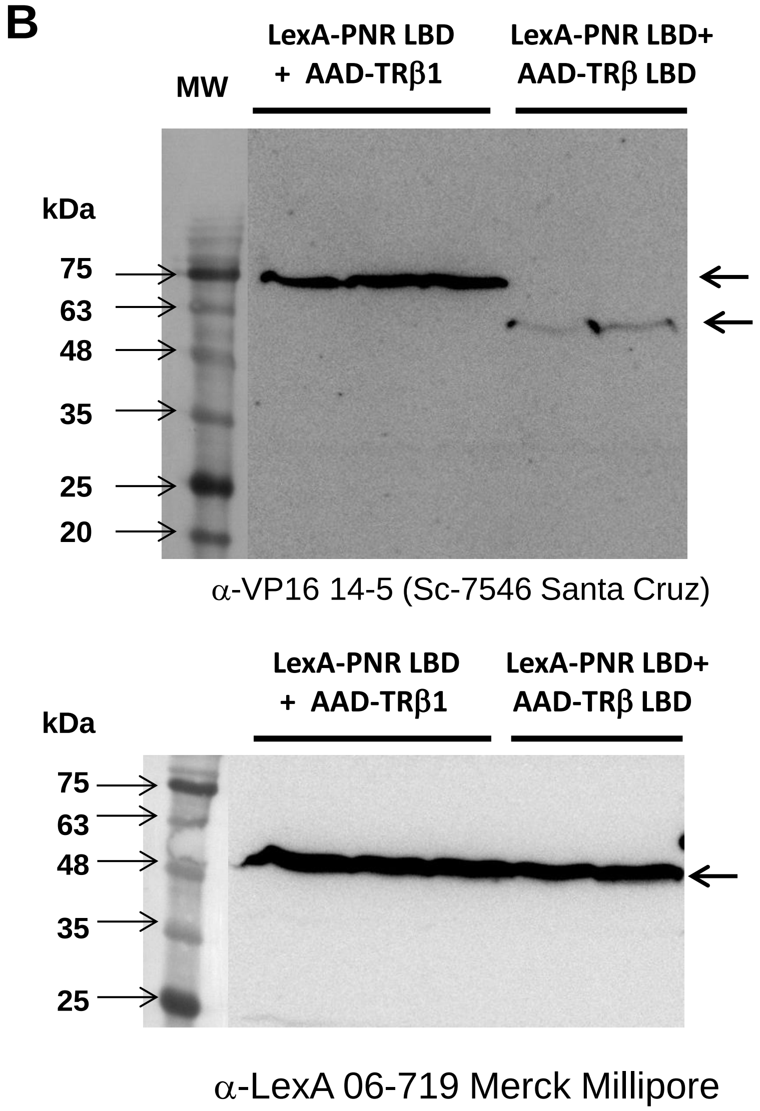
B
MW
LexA-PNR LBD
+ AAD-TRβ1
LexA-PNR LBD+
AAD-TRβ LBD
kDa
75
63
48
35
25
20
α-VP16 14-5 (Sc-7546 Santa Cruz)
LexA-PNR LBD
+ AAD-TRβ1
LexA-PNR LBD+
AAD-TRβ LBD
kDa
75
63
48
35
25
α-LexA 06-719 Merck Millipore

Figure S2

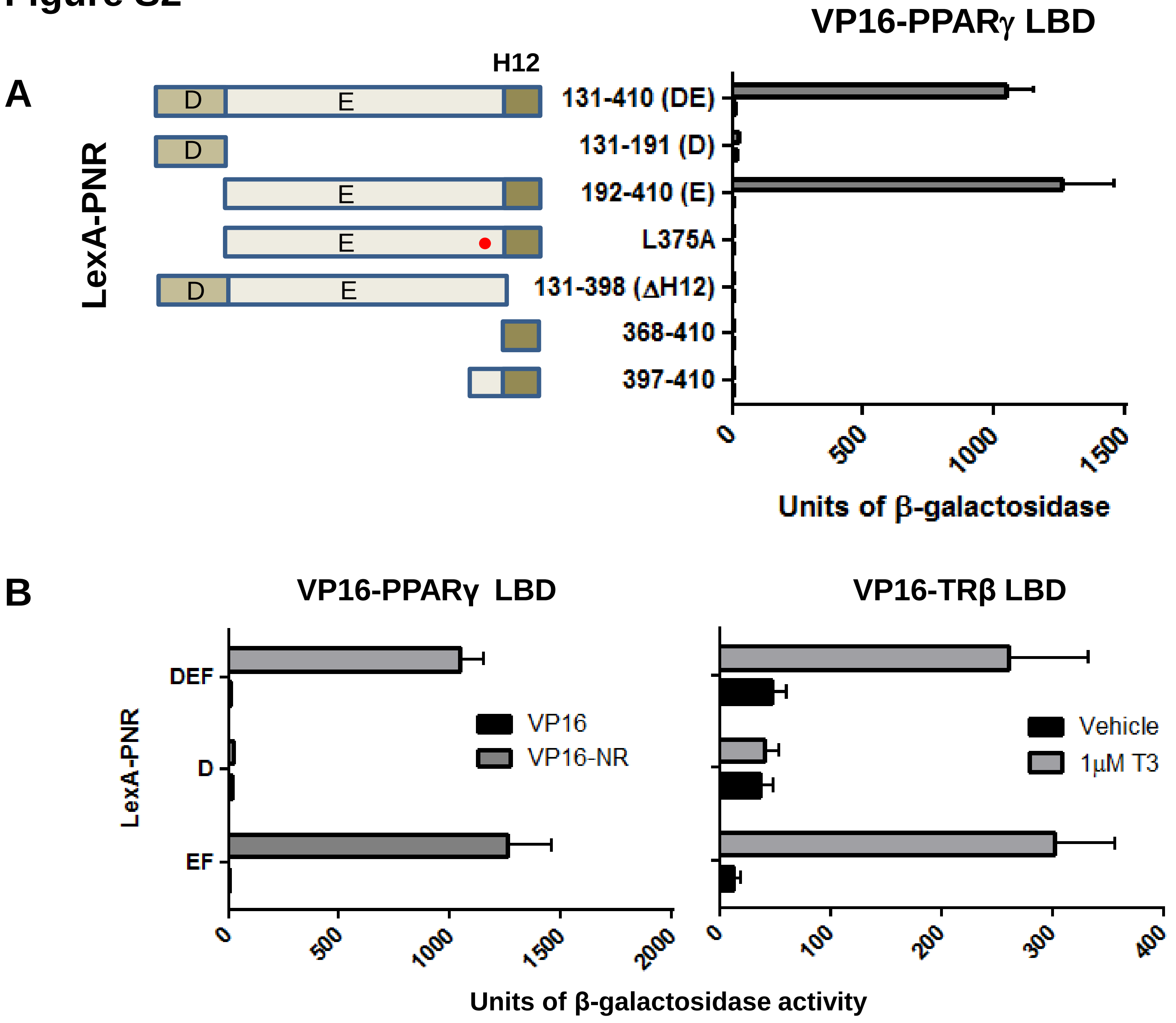

A
VP16-PPARγ LBD
H12
LexA-PNR
D
E
131-410 (DE)
131-191 (D)
192-410 (E)
L375A
131-398 (ΔH12)
368-410
397-410
0
500
1000
1500
Units of β-galactosidase
B
VP16-PPARγ LBD
VP16-TRβ LBD
LexA-PNR
DEF
D
EF
VP16
VP16-NR
Vehicle
1μM T3
0
500
1000
1500
2000
0
100
200
300
400
Units of β-galactosidase activity

Figure S3

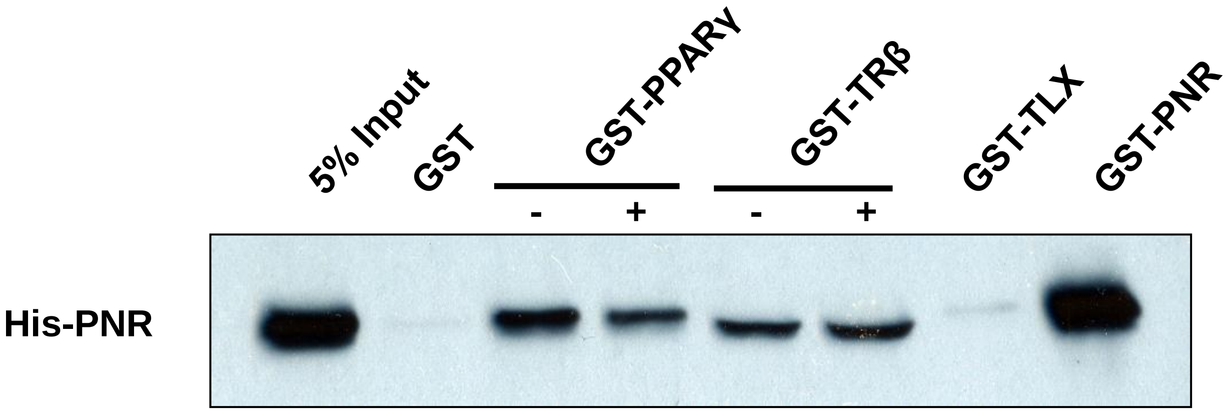

Figure S4

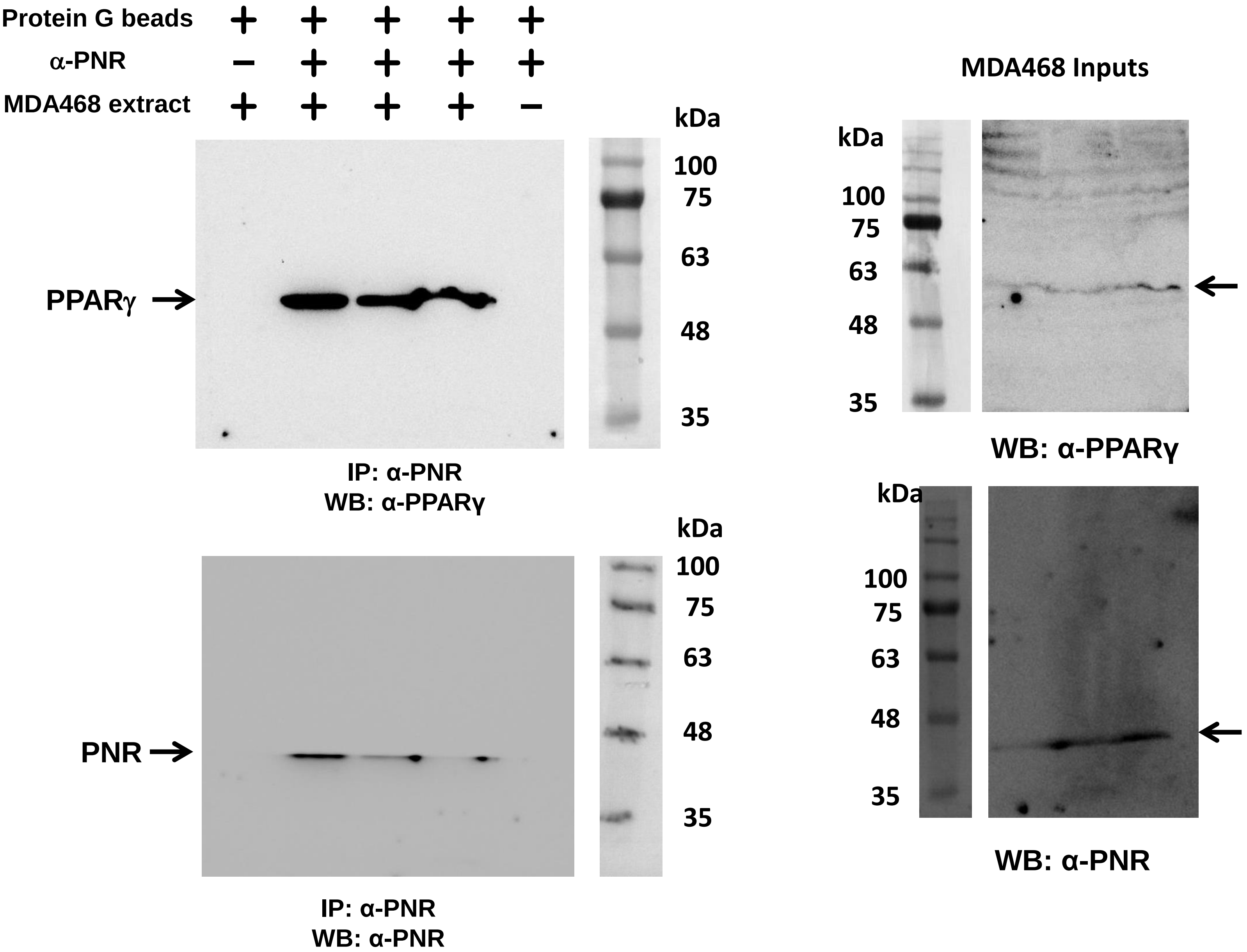

Protein G beads + + + + +
α-PNR − + + + +
MDA468 extract + + + + −
kDa
100
75
63
48
35
PPARγ
IP: α-PNR
WB: α-PPARγ
kDa
100
75
63
48
35
PNR
IP: α-PNR
WB: α-PNR
MDA468 Inputs
kDa
100
75
63
48
35
WB: α-PPARγ
kDa
100
75
63
48
35
WB: α-PNR

Figure S5A

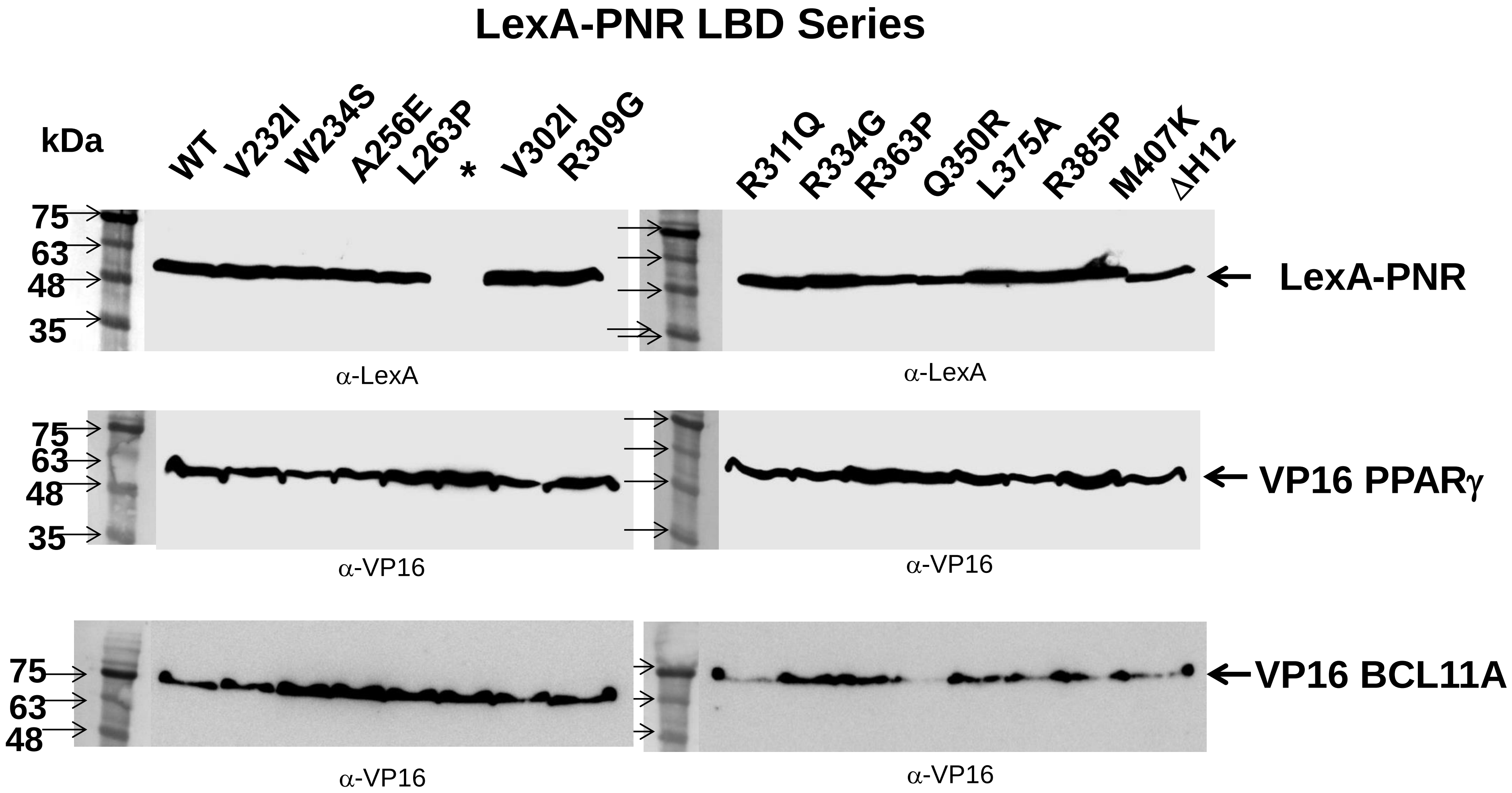
LexA-PNR LBD Series
kDa
WT
V232I
W234S
A256E
L263P
*
V302I
R309G
R311Q
R334G
R363P
Q350R
L375A
R385P
M407K
ΔH12
75
63
48
35
LexA-PNR
α-LexA
α-LexA
75
63
48
35
VP16 PPARγ
α-VP16
α-VP16
75
63
48
VP16 BCL11A
α-VP16
α-VP16

Figure S5B

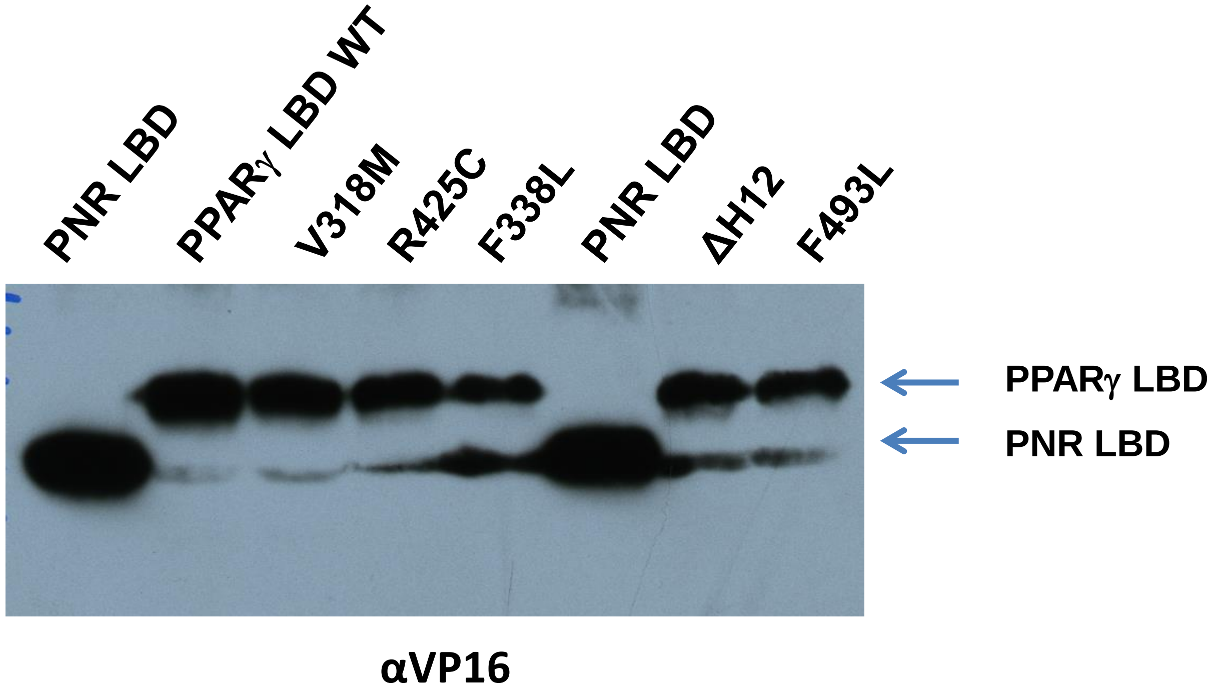
PNR LBD
PPARγ LBD WT
V318M
R425C
F338L
PNR LBD
ΔH12
F493L
PPARγ LBD
PNR LBD
αVP16

Figure S6

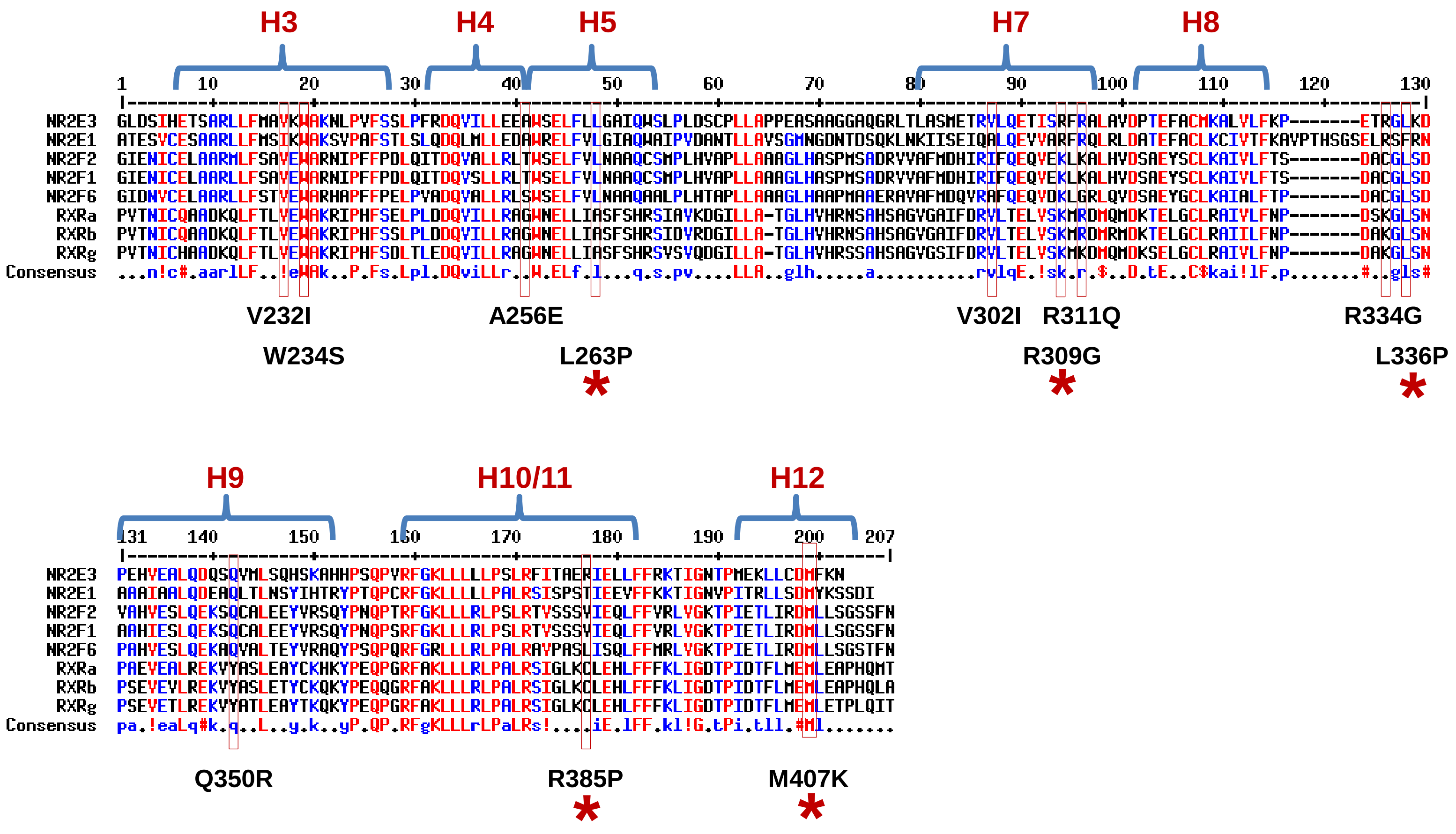
H3
H4
H5
H7
H8
NR2E3
NR2E1
NR2F2
NR2F1
NR2F6
RXRa
RXRb
RXRg
Consensus
V232I
W234S
A256E
L263P
V302I
R311Q
R309G
R334G
L336P
H9
H10/11
H12
Q350R
R385P
M407K

Figure S7

A

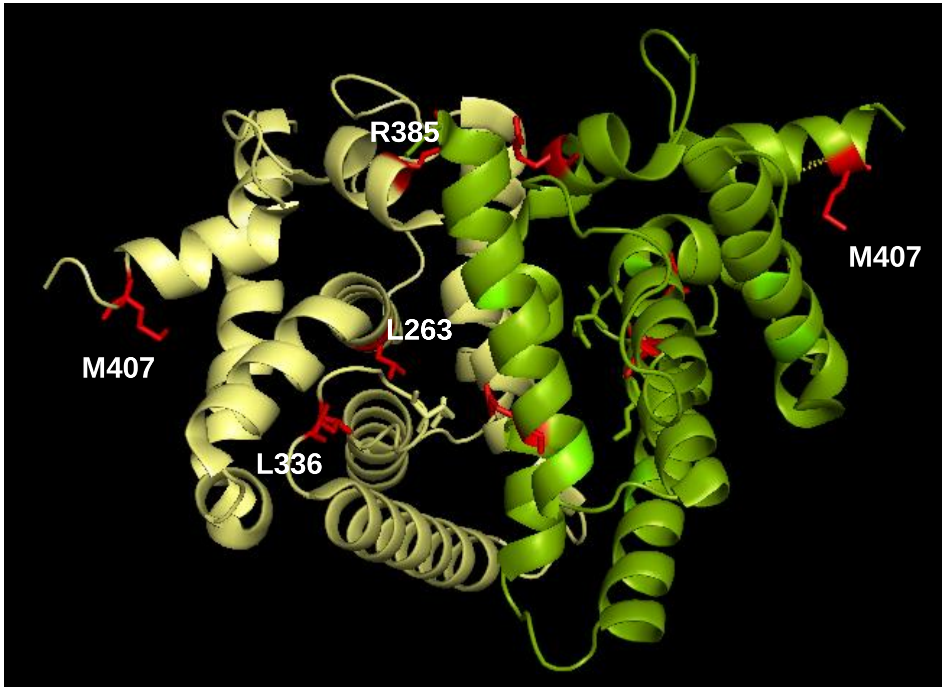

Relative positions of amino acids L263, L336 and R385 In the PNR LBD structure. M407 is located in the AF2 helix.

B

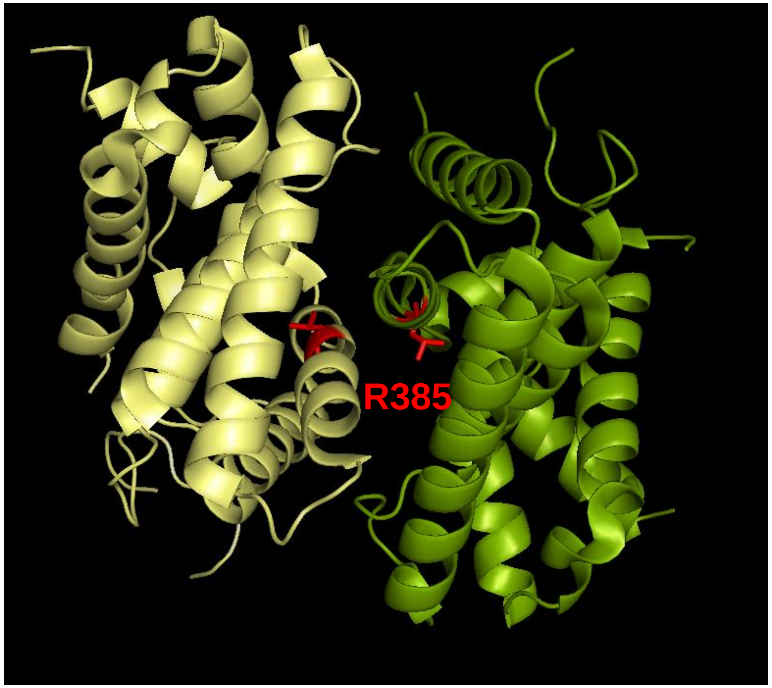

View of R385 (H10) within the PNR LBD homodimer interface

Figure S7

C

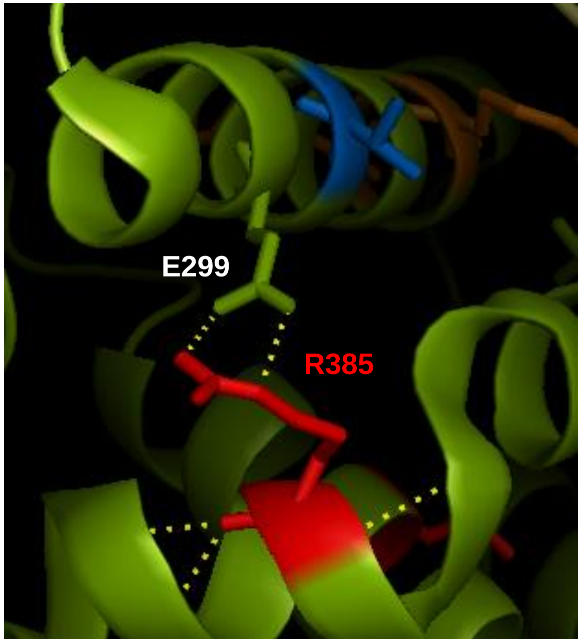

Polar contacts of R385 and E299 (intramolecular)

D

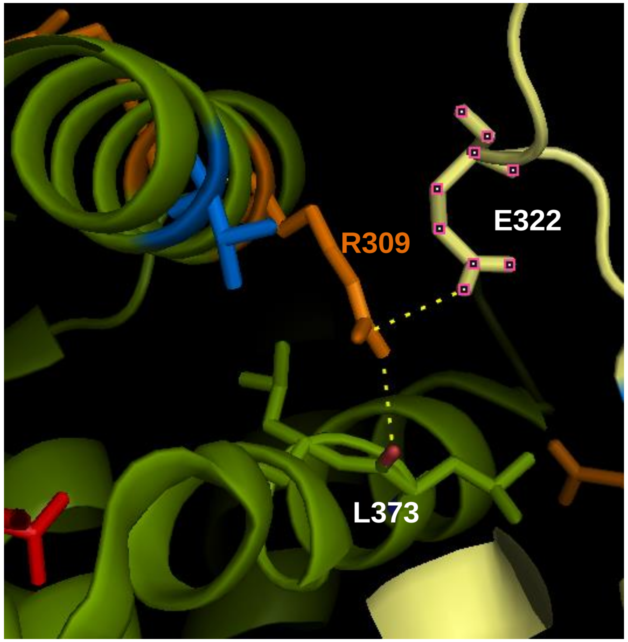

Polar contacts of R309 with the backbone carbonyl of L373 (intra-molecular) and the sidechain of E322 (intermolecular).

E

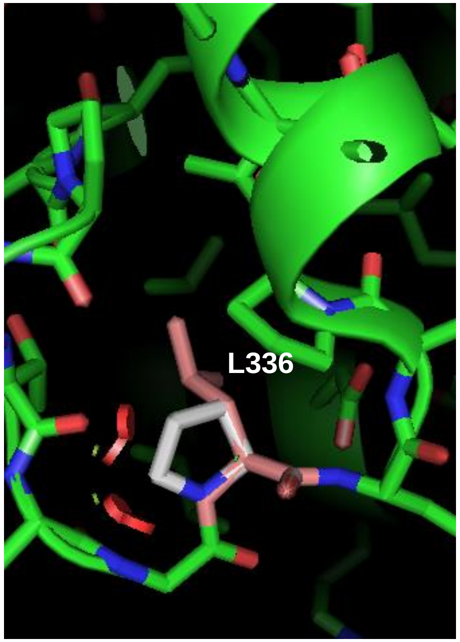

L336 (pink) in the loop between H8 and H9. Mutagenesis to proline ring (grey) introduces steric clashes (red disks).

Figure S8

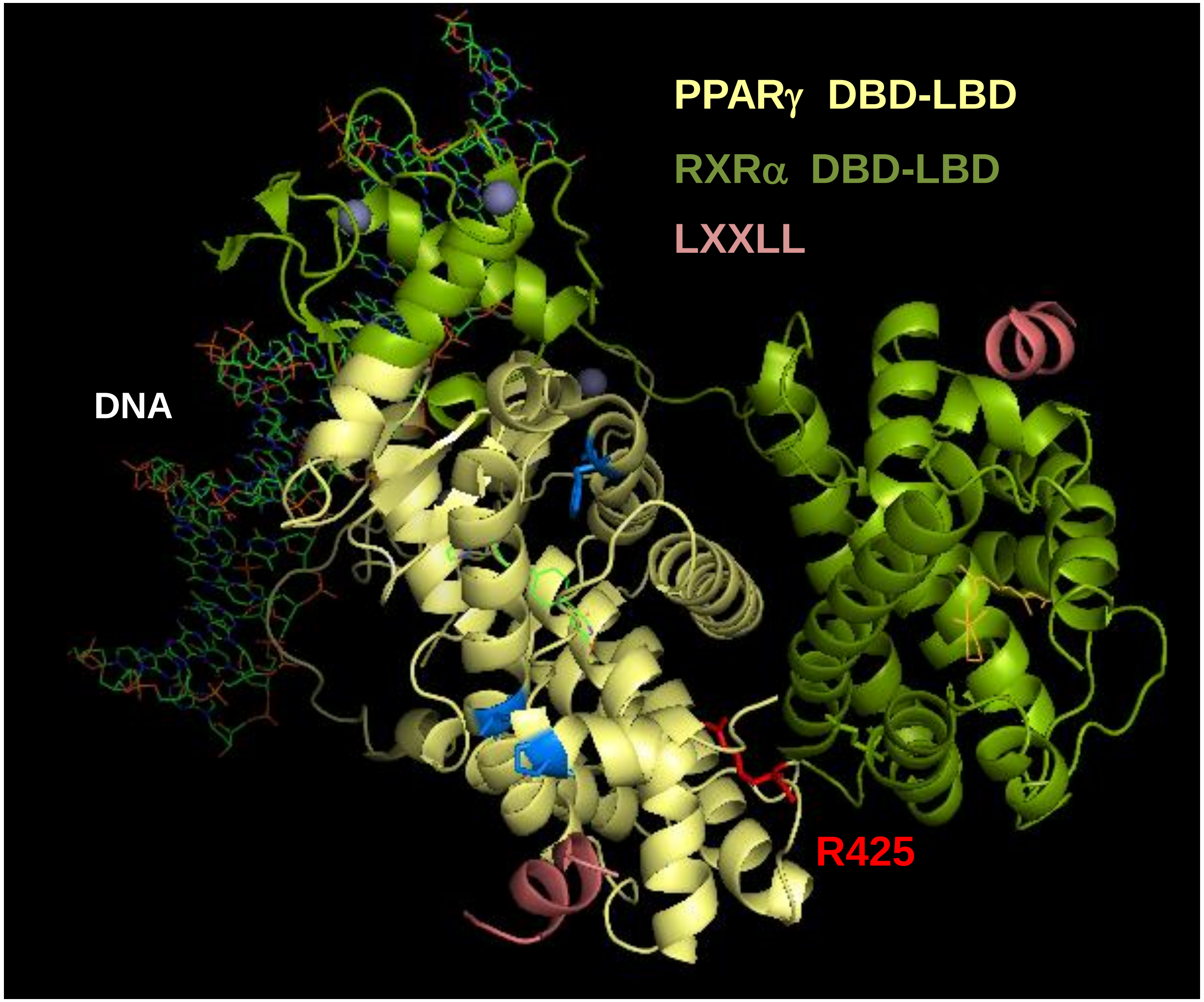

Figure S9

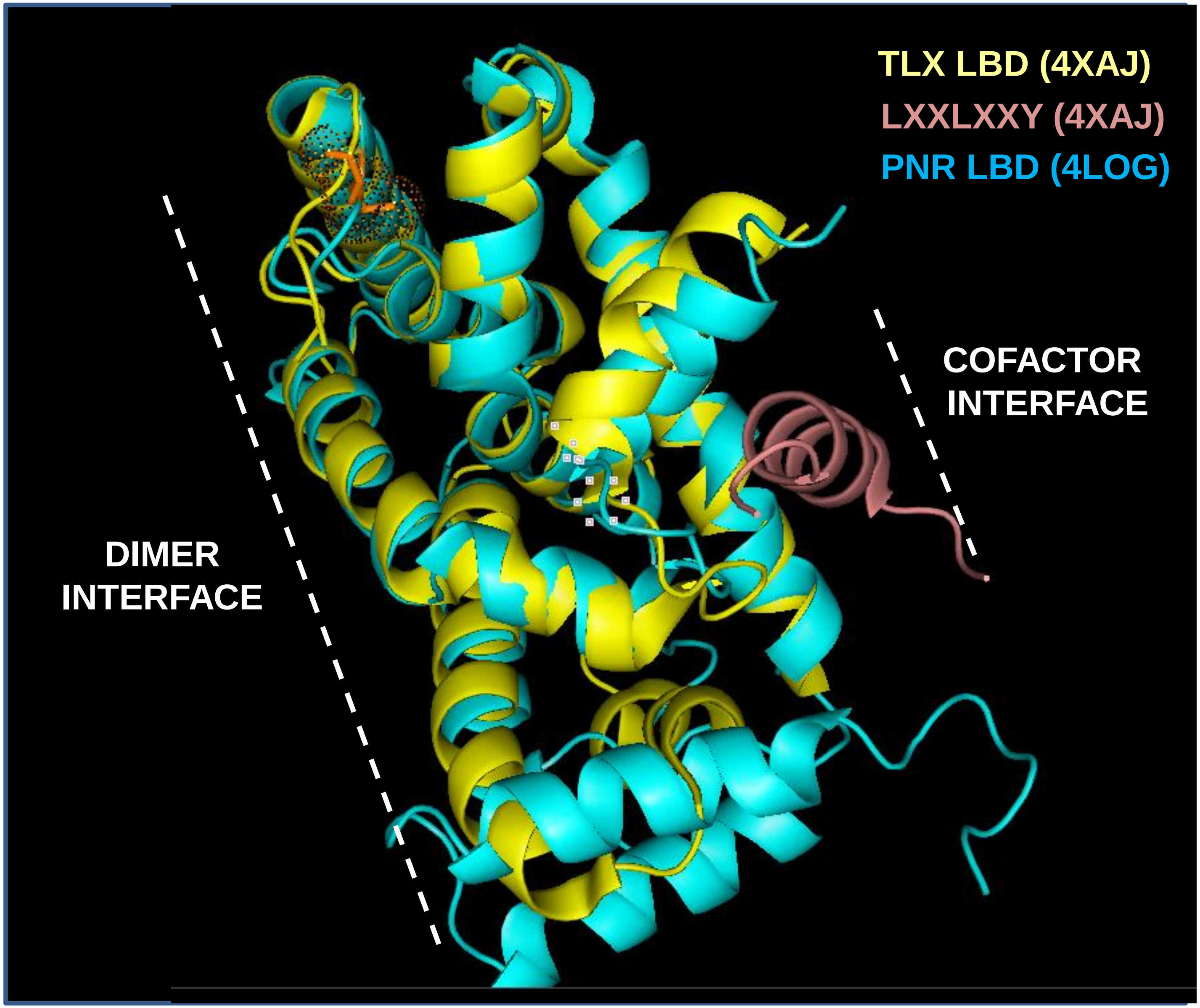
TLX LBD (4XAJ)
LXXLXXY (4XAJ)
PNR LBD (4LOG)
COFACTOR INTERFACE
DIMER INTERFACE
