# Supplementary material for: Heterodimers of photoreceptor-specific nuclear receptor (PNR/NR2E3) and peroxisome proliferator-activated receptor-γ (PPARγ) are disrupted by retinal disease-associated mutations
Source: Cell Death Dis. 2017 Mar 16;8(3):e2677–. doi: 10.1038/cddis.2017.98 (PMC5386588; doi:10.1038/cddis.2017.98)
Supplement: Supplementary Figure Legends [file cddis201798x2.pdf]

Supplementary Figures

Figure S1.

LBD heterodimers of PNR and TRβ

Yeast two-hybrid β-galactosidase reporter assays (A) showing the interaction of LexA-PNR LBD (192-410) and VP16 AAD-TRβ in the presence or absence of Triiodothyronine (T3). The data shown is the mean of three individual transformants, and error bars indicate the standard deviation. (B) Western blots showing detection of VP16-AAD TRβ 6-461 (3 individual transformants) or VP16-AAD-TRβ LBD (2 transformants) with co-expressed LexA-PNR LBD proteins.

Figure S2.

Domain mapping of PNR heterodimer function

Yeast two-hybrid β-galactosidase reporter assays showing (A) the interaction of LexA-PNR regions (as indicated) and with VP16-AAD (black bars) or VP16 AAD-PPARγ-LBD-(222–505) shaded bars. The constructs used in this experiment were LexA-PNR-DEF(131-410) comprising the hinge and LBD, LexA-PNR-hinge-(131-192), LexA-PNR-LBD (192-410), LexA-PNR-LBD (192-410) L375A mutant, LexA-PNR-LBD ΔH12 (192-398) or two constructs comprising the H12 region only, i.e. LexA-PNR (368-410) and LexA-PNR (397-410). (B) Yeast two-hybrid assays assessing the interaction of PNR hinge or LBD constructs with VP16AAD, VP16 AAD-PPARγ-LBD or VP16 AAD TRβ-LBD-(169–461), as indicated. PNR/TRβ interactions were performed in the presence or absence of 1μM T3.

Figure S3.

***In vitro* interactions of full-length PNR with PPARγ and TRβ LBDs**

GST or GST-LBD fusion proteins for the indicated NRs were purified from *E.coli* BL21 and immobilised on glutathione-sepharose beads. Samples were normalised to ensure equal amounts of GST proteins were used. For GST-PPARγ and GST TRβ, binding was performed in the presence of 1μM of the cognate agonist rosiglitazone or T3 (+) or vehicle (-), respectively. Bound proteins were analysed by SDS-PAGE and autoradiography. Input (5%) of *in vitro* translated ^{35}S-labelled His-PNR protein is shown.

Figure S4.

Detection of PNR/PPARγ heterodimer complexes in breast cancer cells.

Breast cancer cell line MDA-MB-468 expresses low levels of PNR and PPARγ, as detected by RT-PCR and western blots. Whole cell extracts were prepared from MDA-MB-468 and incubated with Protein G beads preloaded with anti-PNR antibody. Detection of PNR and PPARγ, proteins in the inputs (10%) are shown in the right-hand panels. After washing of unbound proteins, immuno-purified proteins were recovered and separated by SDS-PAGE. Western blots were performed to detect the immune-purified PNR (predicted MW is 44kDa), and co-immuno-precipitated PPARγ (predicted MW is 55-57kDa) proteins (left hand panels). Controls included Protein G beads with antibody only, or Protein G beads (no antibody) with whole cell extract.

Figure S5.

Expression of PNR and PPARγ LBD wild type and mutant proteins in yeast

(A) Western blots on extracts of yeast L40 transformants expressing LexA-PNR LBD wild type or LBDs containing disease associated variants in combination with VP16-AAD PPARγ LBD or VP16-AAD-BCL11A 1-376. Individual representative clones are shown. The asterix (*) denotes clones containing a LexA-PNR-LBD P276* truncated mutant in combination with the VP16 constructs. Expression of this clone was not detected in this experiment and so it was eliminated from the yeast two-hybrid analysis. (B) Western blots showing detection of VP16-AAD PNR LBD or VP16-AAD PPARγ LBD wild type or mutants proteins in *S.cerevisiae* L40.

Figure S6.

LBD sequence conservation in the NR2E/F and RXR subfamilies

Sequence alignments of the LBDs of the NR2E/F and RXR subfamilies of nuclear receptors. Human reference sequences were obtained from the NCBI database and alignments were performed with Multalin (Corpet, 1988). Genetic variants associated with ESCS analysed in this study are boxed and helices in the LBD are indicated. Variants that are detrimental to dimerization functions of PNR are indicated by a red asterix.

Figure S7. PNR LBD variants associated with ESCS

Structure of the partial PNR LBD (a.a. 217-410) dimeric complex (PDB: 4LOG), which was crystallised as a fusion protein with a N-terminal maltose binding protein

tag (Tan et al., 2013). (A) Relative positions of amino acids L263, L336 and R385 in the PNR LBD structure. M407 is located in the AF2 helix. (B) View of R385 (H10) within the PNR LBD homodimer interface. (C) Polar contacts of R309 with the backbone carbonyl of L373 (intramolecular) and the sidechain of E322 (intermolecular). (D) Polar contacts of R385 and E299 (intramolecular). (E) Modelling of the ESCS–associated variant L336P in the PNR LBD structure. The proline ring side chain is depicted in gray and superposed on the leucine side chain (pink). Partial steric overlap with backbone chain neighbouring residues T333 and R334 is predicted (depicted as red disks). The model was generated using open source PyMol 1.3.

Figure S8. PPARγ LBD mutations in familial partial lipodystrophy

Crystal structure of the PPARγ/ RXRα heterodimer in complex with a PPRE DNA sequence (PDB:3DZY). The PPARγ and RXRα proteins are shown in cartoon representation and consist of the DBD and LBD domains in complex with roziglitazone and 9-*cis* retinoic acid ligands, respectively. Coactivator LXXLL helices are shown in pink. Zinc atoms in the DBDs are shown as grey spheres. DNA chains and ligands are shown as lines. PPARγ residues that are variant in partial lipodystrophy V318, F388, and P495L are represented in blue, whereas R425 is shown in red.

Figure S9

Superimposition of TLX and PNR LBD Structures

Structural alignment of the LBDs of PNR (cyan) and TLX (yellow), (PDB-4XAJ) (Zhi et al., 2015). The position of L336 side chain is represented in orange, and is likely to

impact on the structures participating in NR dimerization interactions. The superimposed position of LXXLXXY motif from the atrophin cofactor that was co-crystallised with the TLX LBD is indicated in pink. Thus sequences participating in cofactor interactions cofactor binding are spatially distant from L336.

References

Corpet, F. (1988). Multiple sequence alignment with hierarchical clustering. Nucleic acids research *16*, 10881-10890.

Tan, M.H., Zhou, X.E., Soon, F.F., Li, X., Li, J., Yong, E.L., Melcher, K., and Xu, H.E. (2013). The crystal structure of the orphan nuclear receptor NR2E3/PNR ligand binding domain reveals a dimeric auto-repressed conformation. PloS one *8*, e74359.

Zhi, X., Zhou, X.E., He, Y., Searose-Xu, K., Zhang, C.L., Tsai, C.C., Melcher, K., and Xu, H.E. (2015). Structural basis for corepressor assembly by the orphan nuclear receptor TLX. Genes & development *29*, 440-450.
