# Supplementary material for: Heterodimers of photoreceptor-specific nuclear receptor (PNR/NR2E3) and peroxisome proliferator-activated receptor-γ (PPARγ) are disrupted by retinal disease-associated mutations
Source: Cell Death Dis. 2017 Mar 16;8(3):e2677–. doi: 10.1038/cddis.2017.98 (PMC5386588; doi:10.1038/cddis.2017.98)
Supplement: Supplementary Material and Methds [file cddis201798x3.docx]

**MATERIALS & METHODS**

Detailed materials and methods for this study

**Expression vectors**

For yeast two-hybrid experiments, PCR fragments were subcloned into plasmids pBTM116mod and pASV3mod (1) to generate LexA DNA binding domain (DBD) and VP16 acidic activation domain (AAD) fusion constructs, respectively. The following AAD-fusion constructs have been described previously; ERα-LBD-(282–595), AR-LBD-(626–919), RARα-LBD-(200–462), RXRα-LBD-(200–462), TRβ-LBD-(169–461), AAD-TRβ-(6-461), PPARγ-LBD-(222–505), VDR-LBD-(123–426), DAX-1-(1–140), SHP-(106–461), Rev-erbβ-LBD-(187–408), RORα-LBD-(106–469), RORβ-LBD-(98–459), HNF4γ-LBD-(88–408), TR2-LBD-(195–467), TR4-LBD-(218–530), TLX-LBD-(100–385), PNR-LBD-(131–410), COUP-TFI-LBD-(151–467), COUP-TFII-LBD-(144–414), EAR2-LBD-(121–399), ERRα-LBD-(169–423), Nur77-(1–599), NURR1-(1–598), LRH-1-LBD-(133–495) and GCNF-LBD-(138–475) (2-4). LexA–BCL11A-(310–325) and LexA-BCL11A-(1-376) have been described previously (4).The following AAD and LexA fusion constructs were generated in this study; AAD-PPARα-LBD-(185–468), AAD-PPARδ-LBD-(157–441), LexA-PNR-LBD-(192-410), LexA-PNR-hinge-(131-192), LexA-PNR (368-410) and LexA-PNR (397-410) and LexA-TLX-LBD-(100-385). PCR-mediated site-directed mutagenesis was used to generate LexA-PNR-LBD-ΔH12 (192-399), LexA-PNR-LBD-(192-410) containing the H10 mutation L375A and the following LexA-PNR-LBD-(192-410) constructs containing mutations encountered in autosomal retinal degenerative conditions; V232I, W234S, A256E, L263P, V302I, R309G, R311Q, R334G, L336P, Q350R, R385P and M407K. We also generated mutations in LexA-PPARγ-LBD-(222-505) associated with lipodystrophies i.e. V318M, F388L, R425C, P495L (numbering in accordance with PPARγ2 isoform: NP_056953.2). Vector cDNAs for PPARα and PPARδ were gifts from Andrew Bennett and Eric Kalkhoven, respectively. The expression of fusion proteins in yeast was monitored by western blotting using antibodies recognizing VP16 AAD (Santa Cruz SC7576) or LexA DBD (Millipore 06-719) as described previously (4).

GST proteins were expressed using a modified version of pGEX-2TK vector (5) and the following constructs were described previously GST-TRβ1-(169-461), GST-COUP-TFI-(151–467), GST-COUP-TFII-(144–414), GST-PNR-(89–410) and GST-TLX-(100–385) (3, 4). The construct pGEX-2TK-PPARγ-LBD-(173–475) was a gift from E. Kalkhoven. The mammalian cell expression plasmid pcDNA3.1 HA-PNR and pCH110-lacZ were described previously (4); pcDNA3.1/His-PNR-(1-410) contains an N-terminal hexa-histidine tag and was a gift from Shiming Chen. His-PNR-(1-410) R309G were generated by site-directed mutagenesis. Flag-PPARγ was purchased from Addgene and reporter plasmid p(PPRE)3-tk-Luc was a gift from Eric Kalkhoven. All constructs generated by PCR or site-directed mutagenesis were sequenced to confirm their validity.

**Yeast two-hybrid interaction assays**

*Saccharomyces cerevisiae* L40 [trp1, leu2, his3, ade2,LYS2::(lexAop)4x-HIS3, URA3::(LexAop) 8x-LacZ] was co-transformed with LexA-fusion and AAD-NR-fusion expression vectors using the lithium acetate method as described previously (4). Single transformants containing the desired plasmids were selected on appropriate media and grown to late log phase in 15 ml of selective medium (yeast nitrogen base containing 2% w/v glucose and appropriate supplements) in the presence of 10^−6^ M cognate ligand or vehicle. Preparation of cell-free extracts was by the glass bead method, and β-galactosidase assays were performed as described (1). Reporter β-galactosidase activities in the presence or absence of ligand (where known) were determined for three individual transformants for each condition, in replicated experiments as stated. Ligands for RARα (all-trans retinoic acid), RXRα (9-cis retinoic acid), ERα (17-β-estradiol), AR (mibolerone), PPARγ (rosiglitazone), TRβ (triiodothyronine) and VDR (cholecalciferol) were purchased from Sigma. Western blots were used to confirm expression levels of LexA-DBD and VP16-AAD fusion proteins, as described previously (4).

**GST pull-down assays**

GST fusion proteins were expressed in *E. coli* BL21 or Rosetta using isopropyl-β-D-1 thiogalactopyranoside induction, and purified on glutathione-Sepharose beads (Amersham Biosciences). The pcDNA3.1 His-PNR and PNR R309G were *in vitro* transcribed/translated in the presence of [35S]-methionine in reticulocyte lysate (Promega) according to the manufacturer’s instructions. Equalised amounts of GST proteins were incubated with 35S-radiolabelled protein in NETN buffer (20mM Tris, pH 8.0, 100mM NaCl, 1mM EDTA, 0.5% NP-40) containing 1X complete protease inhibitors (Roche Molecular Biochemicals) in the presence or absence of 10^−6^ M cognate ligand as described previously (2). Samples were washed three times, and bound proteins were separated by SDS-PAGE. Radio-labelled proteins in dried gels were visualized by autoradiography.

**Cell culture, transient transfections, reporter assays**

U2OS, HEK293 and MDA468 cells were cultured in DMEM supplemented with 10% fetal bovine serum (FBS) and maintained as described previously (5-7). Twenty-four hours prior to transfection, U2OS cells were reseeded in phenol red-free DMEM supplemented with 5% dextran charcoal-stripped FBS. Transient transfections were performed using calcium phosphate co-precipitation. For reporter assays, transfected DNA included pCH110-lacZ internal control plasmid (500 ng/well), p(PPRE)3-tk-Luc (100 ng) luciferase reporter plasmid and varying amounts of pcDNA3.1 containing His-PNR, His-PNR R309G and Flag-PPARγ as indicated. Empty pcDNA3.1 vector was used to standardize the amount of transfected DNA. After 16 h, fresh medium containing either 10^−7^M rosiglitazone (Ros) or vehicle was added. After a further 24 h, cells were harvested and cell-free extracts were prepared and assayed for luciferase activity using the Dual light® System (Applied Biosystems) and normalised to β-galactosidase activities. Reporter assays were performed in triplicate.

**Immunoprecipitation and western blots**

To assess co-immunoprecipitation of endogenous PNR, TLX and PPARγ proteins, retinal tissue extract was prepared (8) using donor tissue (fresh posterior segments free of known ocular disease) sourced from Manchester Eye Bank, undertaken with approval of the local research ethics committee (Univ. Nottingham, Q1060301). The retina was dissected and cut into 1mm pieces and washed with sterile phosphate-buffered saline. Nuclear extracts of tissue homogenates were prepared and subject to immunoprecipitation as described previously (4). The precipitates were resolved by SDS-PAGE for Western blot analysis. Immunoprecipitations were performed using anti-human PNR/NR2E3 (H7223; R&D Systems) or IgG control. Western blots were performed using anti-PNR (H7223); anti-TLX/NR2E1 (S-23; sc-133843 Santa Cruz) and anti-PPARγ (H100 Sc-7196; Santa Cruz).

Co-immunoprecipitation of NR protein complexes in MDA-MB-468 cells was performed as follows: cells at 80% confluence were harvested and resuspended in lysis buffer (20 mM sodium phosphate [pH 7.0], 250mM NaCl, 30mM sodium pyrophosphate, 0.1% Nonidet P-40, 5mM EDTA, 10mM NaF, 0.1mM Na3VO4, 1mM phenylmethylsulfonyl fluoride) supplemented with 1mg of leupeptin per ml, 1mg of pepstatin per ml, and 1mg of aprotinin per ml. Magnetic Protein G beads were washed in lysis buffer and loaded with 1μg anti-PNR Sc-374513 for 1 hour at room temperature. Subsequently, 800 μg of protein in a total volume of 0.5ml lysis buffer was applied. Following overnight incubation with rotation at 4^o^C, supernatant was removed, beads were washed 3 times in lysis buffer and resuspended in loading dye before analysis by SDS PAGE. After blotting, immunoprecipitated bands were revealed using anti-PPARγ (E8) Sc-7273 and anti-PNR (B4) Sc-374513 in combination with a chicken anti-mouse secondary antibody Sc-2954.

**Electrophoretic mobility shift assay**

Forward and reverse strands of nucleic acid probes were subjected to 5′-hydroxyl end labelling with [γ32P]-ATP, using T4 polynucleotide kinase. The labelled probes were annealed and purified on G-25 sephadex columns, before being adjusted to 100,000 cpm/μl for use in binding assays. The sequence of the DR1 (PPRE) sequence was as follows, 5’-GGTAAAGGTCAAAGGTCAAT-3’. Nuclear extracts were prepared from HEK293 cells transfected with pcDNA3.1-His-PNR, His-PNR-R309G, HA-TLX, Flag-PPARγ or RXRα expression vectors. Expression of the recombinant proteins in nuclear extracts was verified by western blots. Equal amounts of NE for different conditions were incubated with 1 µl nucleic acid probe, 1 μg poly(dI:dC)·(dI:dC) and 3 μg bovine serum albumin in electrophoretic mobility shift assay (EMSA) buffer (20 mM HEPES-KOH pH 7.9, 12% v/v glycerol, 50 mM KCl and 1 mM DTT) to a final volume of 10 µl. Binding mixes were incubated at room temperature for 20 min to allow DNA/protein complex formation. Where appropriate, specific antibodies for FLAG (A2220, Sigma) or PNR (ab41922, Abcam) were added to binding mixes to validate the identity of protein/DNA complexes. Binding mixes were loaded onto 5% polyacrylamide gels and electrophoresed in 0.5× TBE prior to being visualized by autoradiography.

1. Le Douarin B, Heery DM, Gaudon C, vom Baur E, Losson R. Yeast two-hybrid screening for proteins that interact with nuclear hormone receptors. Methods in molecular biology. 2001;176:227-48.

2. Heery DM, Kalkhoven E, Hoare S, Parker MG. A signature motif in transcriptional co-activators mediates binding to nuclear receptors. Nature. 1997;387(6634):733-6.

3. Coulthard VH, Matsuda S, Heery DM. An extended LXXLL motif sequence determines the nuclear receptor binding specificity of TRAP220. The Journal of biological chemistry. 2003;278(13):10942-51.

4. Chan CM, Fulton J, Montiel-Duarte C, Collins HM, Bharti N, Wadelin FR, et al. A signature motif mediating selective interactions of BCL11A with the NR2E/F subfamily of orphan nuclear receptors. Nucleic acids research. 2013;41(21):9663-79.

5. Sheppard HM, Harries JC, Hussain S, Bevan C, Heery DM. Analysis of the steroid receptor coactivator 1 (SRC1)-CREB binding protein interaction interface and its importance for the function of SRC1. Molecular and cellular biology. 2001;21(1):39-50.

6. Collins HM, Kindle KB, Matsuda S, Ryan C, Troke PJ, Kalkhoven E, et al. MOZ-TIF2 alters cofactor recruitment and histone modification at the RARbeta2 promoter: differential effects of MOZ fusion proteins on CBP- and MOZ-dependent activators. The Journal of biological chemistry. 2006;281(25):17124-33.

7. Kindle KB, Troke PJ, Collins HM, Matsuda S, Bossi D, Bellodi C, et al. MOZ-TIF2 inhibits transcription by nuclear receptors and p53 by impairment of CBP function. Molecular and cellular biology. 2005;25(3):988-1002.

8. Stewart EA, Samaranayake GJ, Browning AC, Hopkinson A, Amoaku WM. Comparison of choroidal and retinal endothelial cells: characteristics and response to VEGF isoforms and anti-VEGF treatments. Experimental eye research. 2011;93(5):761-6.
